# Seed-mediated atomic-scale reconstruction of silver manganate nanoplates for oxygen reduction towards high-energy aluminum-air flow batteries

**DOI:** 10.1038/s41467-018-06211-3

**Published:** 2018-09-13

**Authors:** Jaechan Ryu, Haeseong Jang, Joohyuk Park, Youngshin Yoo, Minjoon Park, Jaephil Cho

**Affiliations:** 1 Department of Energy Engineering, School of Energy and Chemical Engineering, Ulsan National Institute of Science and Technology (UNIST), 50, UNIST-gil, Ulsan, 44919 Republic of Korea; 2 Present Address: Department of Materials, Parks Road, University of Oxford, Oxford, OX1 3PH UK

## Abstract

Aluminum–air batteries are promising candidates for next-generation high-energy-density storage, but the inherent limitations hinder their practical use. Here, we show that silver nanoparticle-mediated silver manganate nanoplates are a highly active and chemically stable catalyst for oxygen reduction in alkaline media. By means of atomic-resolved transmission electron microscopy, we find that the formation of stripe patterns on the surface of a silver manganate nanoplate originates from the zigzag atomic arrangement of silver and manganese, creating a high concentration of dislocations in the crystal lattice. This structure can provide high electrical conductivity with low electrode resistance and abundant active sites for ion adsorption. The catalyst exhibits outstanding performance in a flow-based aluminum–air battery, demonstrating high gravimetric and volumetric energy densities of ~2552 Wh kg_Al_^−1^ and ~6890 Wh l_Al_^−1^ at 100 mA cm^−2^, as well as high stability during a mechanical recharging process.

## Abstract (web summary)

Aluminum-air batteries are lightweight and cost effective, but performance is limited by corrosion and solid by-products. Here the authors catalyze oxygen reduction with silver manganate nanoplates and develop an aluminum-air flow battery that delivers high energy density and alleviates side reactions.

## Introduction

Metal–air cells have been highlighted as next-generation batteries, providing the possibilities of low cost and high-energy storage^1^. Aqueous aluminum–air batteries (AABs) are a promising candidate for efficient power delivery in the field of transportation and uninterrupted power supply because of its high-energy density (theoretically, ~8131 W h kg_Al_^−1^ and ~21,954 W h L_Al_^−1^_,_ see Supplementary Note 1) with abundant reserves, low cost, and light weight of aluminum (26.98 g mol^−1^)^2,3^. In particular, aluminum has the highest volumetric capacity (~8.04 A h cm^−3^) among the electrode materials (e.g., Li: ~2.06, Mg: ~3.83, and Zn: ~5.85 A h cm^−3^)^4^. However, there are several hurdles that need to be overcome for the practical application of the AABs, such as high corrosion rate of aluminum anodes, rapid self-discharge, and precipitation of solid by-products (Al(OH)_3_ and Al_2_O_3_) on the surface of electrodes^5–7^. Thus, technical progress has been mainly limited to the mismatch between theoretical and practical performances (< 1000 W h kg_Al_^−1^)^8,9^. To date, intensive efforts have been devoted to overcome the above-mentioned issues by developing a proper aluminum alloy as anodes^10–12^, electrolytes^13–15^, and cell designs^16,17^. Recently, AABs with Cu/Cu_2_O nanoparticles and Cu–N–C with the Ketjenblack supports were reported by using a sacrificial Cu-based metal–organic framework, showing a stable discharge of 1.53 V at 40 mA cm^−2^ with the aid of additives^18^. In terms of the cell design, AABs containing two different electrolytes were also reported^15^ and demonstrated a specific discharge capacity of ~1810 mA h g_Al_^−1^ and energy of ~2081 W h kg_Al_^−1^ at a current density of 20 mA cm^−2^. In addition, Shim et al. fabricated deformable AABs with the high degree levels, which exhibited a specific capacity of 496 mA h g_Al_^−1^ at a current density of 5 µA cm^−2^.^19^ However, its poor rate capability with low current density still limited the practical use. Thus, it is urgent to develop highly efficient, feasible alternative approaches. There is an additional intrinsic problem in AABs, which is a sluggish rate of the oxygen reduction reaction (ORR). Research into electrocatalyts for ORR can be categorized into metal- and carbon-based materials^20–32^. With respect to the lifetime of an air electrode, the carbon could facilitate the formation of hydroperoxide during ORR^33^; thus, carbon-free metal or metal oxides have been widely studied^34–38^. Manganese oxides have emerged as an efficient electrocatalyst for ORR,^39–41^ showing high catalytic activity according to their chemical structures and morphologies such as nanowires^42,43^, nanorods^44,45^, and nanoplates^46^. In particular, plate-like manganese oxides have attracted tremendous research interest because of their unique physiochemical properties with well-defined interfaces, which have been applied to a range of electrochemical devices^47^. However, typical oxides have low electrical conductivity, which can be enhanced by introducing highly conductive precious metals such as platinum^48^, palladium^49^, or silver^30,50,51^. Among them, silver has several appealing features, such as approximately 50 times lower cost and less susceptibility in alkaline solution in comparison to platinum^34^. Currently, several silver manganese oxide materials and alloys have been reported, for example, Ag–MnO_x_ nanowire^50^, Ag–MnO_2_ on reduced graphene oxide^51^, and Ag–MnO_x_ carbon composite^52^. However, a critical question remains with regard to how the silver element can improve the catalytic activity of manganese oxide in terms of the atomic structure.

In this study, we demonstrate the incorporation of silver atoms on a plate-like manganese oxide via a metal seed-mediated growth method. This enables the formation of a striped silver manganate structure with abundant surface dislocations, leading to high catalytic activity and electron transfer rate for ORR. Additionally, we develop a flow-based aluminum–air battery to alleviate the side reactions in the cell, where the electrolytes can be continuously circulated. Remarkably, the precipitation problem has been addressed, achieving the unprecedented energy density of ~2552 W h kg_Al_^−1^ (~6861 W h L_Al_^−1^) at a high current density of 100 mA cm^−2^.

## Results

### Electrocatalyst preparation and characterization

**Fig. 1:**
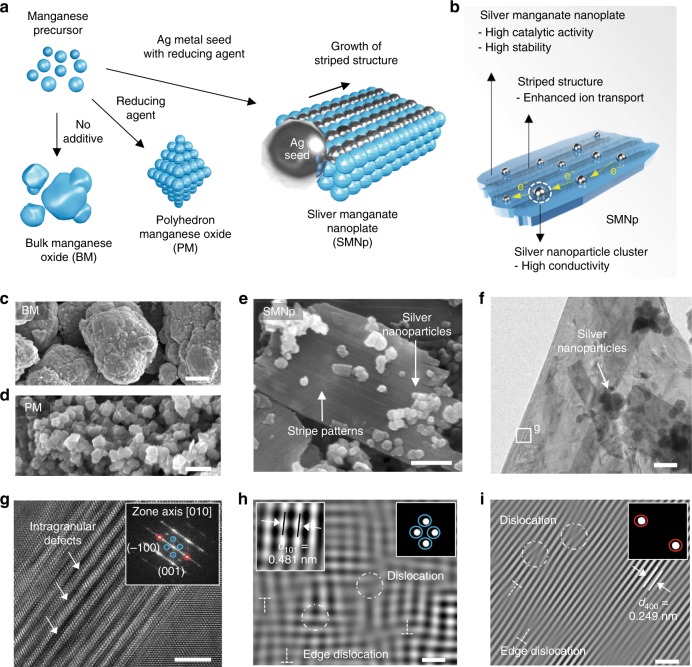
Formation of the stripe pattern and dislocation. **a**, **b** Schematic of the synthesis process (**a**) of the bulk manganese oxides (BM), polyhedron manganese oxide (PM), and silver manganate nanoplate (SMNp), and the summary of the catalytic effects (**b**) of the SMNp. **c**–**e** Scanning electronic microscopy (SEM) images of the BM (**c**), PM (**d**), and SMNp (**e**) showing plate-like structures with silver nanoparticles. **f**, **g** High-resolution transmission electron microscopy (HR-TEM) images of the SMNp (**f**) and high-magnified image (**g**) of the box in **f**, which shows zigzag atomic arrangements with intragranular cracking. Fast Fourier transform (FFT) image of **g** presents the lattice planes of (–100) and (001) along the [010] zone, indicating the orthorhombic structure of Ag_2_MnO_4_. **h**, **i** Inverse FFT images of applied masks at the blue circle (**h**) and red circle (**i**) in **g**, showing a wide range of dislocations with defects. The dislocations are shown by circles and edge dislocations are represented by a T-shaped symbol. The upper right insets in **h** and **i** indicate the applied masks and the lattice spacing of the (101) plane is denoted as the left inset in **h**. Scale bars, 5 μm (**c**), 500 nm (**d**), 200 nm (**e**), 100 nm (**f**), 4 nm (**g**), and 1 nm (**h**, **i**)

Our unique strategy enables the formation of silver metal seed-mediated silver manganate nanoplates (hereafter referred to as SMNp) by simple precipitation and heat-treatment methods. Briefly, the manganese precursor and silver nanoparticles functionalized with polyvinylpyrrolidone as a chelating agent were dispersed in ethanol solution, and then NaOH as a reducing agent was added under stirring. After the precipitation reaction was completed, the prepared manganese hydroxide with a silver nanoparticle was calcined at 450 °C for 15 min (see Methods). Figure 1a shows the schematic of different morphologies of each sample according to synthesis conditions. For comparison, bulk manganese oxide (BM) and polyhedron manganese oxide (PM) were prepared without a reducing agent or silver metal seed, respectively. One observation is that the incorporation of silver metal in a manganese hydroxide precursor enabled the growth of the plate-like SMNp, in which silver nanoparticles could act as a growth seed (Supplementary Fig. 1). The lower coordination of surface atoms (typical low-index (111) or (100)) was reported to induce the internal lattice strain to minimize the surface energy^46^. In our case, the silver nanoparticles in the SMNp, which has a low index of (111) and a particle size of < 100 nm are expected to induce the similar structural disorder, thus generating surface strain in MnO_x_.

In addition, the unique striped patterns with intragranular defects were observed in the SMNp, which could be ascribed to the zigzag atomic arrangements of silver and manganese elements. This result implied that as growth proceeded during heat treatment, the silver atom could move to the available site on the manganese substrate by creating the orthorhombic crystal structure of Ag_2_MnO_4_. Figure 1b summarized the catalytic effect of the SMNp in terms of the electrochemical properties, which will be discussed in detail.

The scanning electron microscopy (SEM) of the BM shows that the particle shape was bulky agglomerates with a size of ~15 µm because of the lack of additives for the morphology control (Fig. 1c), and X-ray diffraction (XRD) pattern indicated that it contained different phases of α-Mn_2_O_3_ and β-MnO_2_ (Supplementary Fig. 2a). In contrast, the PM showed nano-sized polycrystalline structure with a size of ~100 nm (Fig. 1d), and consisted of mixed Mn_3_O_4_ and Mn_5_O_8_ phases (Supplementary Fig. 2b). We used a crystalline silver metal seed having an average size of ~100 nm, which is typically enclosed by four facets, for example, (111), (200), (220), and (311), as shown in Supplementary Fig. 2c. By using this metal seed, the plate-like configuration of the SMNp was obtained with distributed silver nanoparticles on the surface (Fig. 1e), showing a thickness of 30–40 nm (Supplementary Figs. 3, 4). Energy-dispersive X-ray spectroscopy (EDX) mapping revealed that the SMNp was composed of the silver, manganese, and oxygen atoms (Supplementary Fig. 5), and XRD analysis also exhibited four different phases of Ag, Ag_2_O, Ag_2_MnO_4_, and Mn_3_O_4_ (Supplementary Fig. 2d). This result was consistent with the chemical position data from X-ray photoelectron spectroscopy (XPS) analysis. The Ag 3*d*, Mn 2*p,* and O 1*s* core levels from the SMNp structure exhibited the pronounced peaks related to the Ag_2_O and Mn_3_O_4_ at 367.5, 373.5, 529.7, and 641.7 eV (Supplementary Fig. 6). We also measured the specific surface areas of the BM, PM, and SMNp, showing the values of 4.97, 14.18, and 11.47 m^2^ g^–1^, respectively, where the PM showed the highest value because it consisted of the nanoparticles (Supplementary Table 1).

High-resolution transmission electron microscopy (HR-TEM) image of the SMNp clearly showed the striped patterns on the surface along with a wide range of dislocations and intragranular defects (Fig. 1f, g). The fast Fourier transform (FFT) image of the SMNp showed the orthorhombic phases on the basis of the unit spots of (−100) and (001) along the [010] zone axis, which are consistent with XRD results of Ag_2_MnO_4_ (space group: Pnma). This implies that the structure of nanoplate parts in the SMNp was Ag_2_MnO_4_ and not manganese oxide itself. To understand the complex structures of the SMNp, inverse FFT images were obtained by applying different masks on it along the [010] zone axis (Fig. 1h, i). Apparently, a high concentration of dislocations with crystal defects was discovered on the striped structures of the SMNp, and the other zone axis of [123] direction also showed similar results. Notably, from the dislocation edges, d-spacing value was increased from ~0.389 to ~1.124 nm, indicating continuously propagated defects (Supplementary Fig. 7). These dislocation edges could act as the nucleation sites, generating the intragranular cracks^53^. The evolution of dislocation could be originated from the different atomic size of silver and manganese elements within the crystal lattice. The larger atomic size of the silver element than the manganese element could trigger a substantial lattice strain in the nanoplate, which significantly dislodges nearby atoms and generates an anisotropic defect structure in the SMNp^54^. In terms of catalytic activity, the exposed defects may provide abundant active sites and ionic channels, reinforcing the ion transport kinetics. To clarify the morphology change and the concentration of dislocations of the SMNp, the control experiment with different amounts of precursors (silver nanoparticle, manganese source, and reducing agent) was conducted (see Methods), which was summarized in Supplementary Figs. 8–10. As a result, increasing the amounts of silver nanoparticles reduced the concentration of dislocations with lower defects, showing high crystallinity of selected area electron diffraction (SAED) patterns (Supplementary Fig. 8). On the contrary, nanoplate morphology was not observed when both amounts of manganese and the reducing agent were controlled (Supplementary Figs. 9, 10). This implied that the seed-mediated growth for forming nanoplate morphology required appropriate contents of manganese source and the reducing agent.

**Fig. 2:**
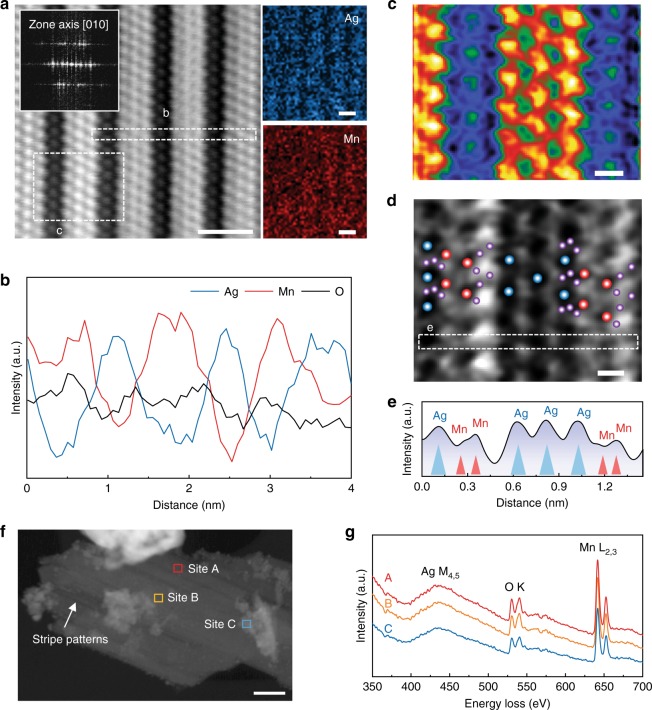
Atomically resolved electron microscopy and electron energy loss spectra. **a** Filtered high-angle annular dark-field-scanning transmission electron microscopy (HAADF-STEM) and energy-dispersive X-ray spectroscopy (EDX) mapping image of the silver manganate nanoplate (SMNp) along the [010] zone axis with its fast Fourier transform (FFT) pattern, showing continuous arrangements of silver and manganese atoms. **b** Line-scanning profile showing distinct distributions of silver and manganese elements. **c**, **d** Simulated HAADF-STEM image (**c**), indicating the highest electron density with red color and the lowest with black, and annular bright-field (ABF)–STEM image (**d**) showing the Ag_2_MnO_4_ orthorhombic structure with silver (blue), manganese (red), and oxygen (purple) elements. **e** Corresponding ABF intensity profiles of the white box in **d** present the peaks of the silver and manganese atomic columns. **f**, **g** A HAADF-STEM image (**f**) and the corresponding electron energy loss spectroscopy (EELS) profiles (**g**) at the marked sites, showing Ag M_4,5_-edge, O K-edge, and Mn L_2,3_-edge. Scale bars, 1 nm (**a**), 0.2 nm (**c**, **d**), and 50 nm (**f**)

To further confirm the origin of striped pattern on the SMNp, we performed atomically resolved scanning transmission electron microscopy (STEM) and electron energy loss spectroscopy (EELS). To clearly track the silver and manganese arrangements, we used high-angle annular dark-field STEM (HAADF-STEM) analysis and STEM–EDX mapping of the SMNp. As shown in Fig. 2a, individual atomic columns of silver and manganese were well resolved along the [010] zone direction, and STEM–EDX line-scanning profile provided distinct fluctuating curves of silver and manganese atoms with well-distributed oxygen elements (Fig. 2b). The simulated HAADF-STEM showed the sequence of atomic rows with different electron densities, further confirming that the striped structure was caused by silver and manganese arrangements (Fig. 2c). More striking was the detection of oxygen sites near the silver and manganese atoms by annular bright-field STEM (ABF-STEM) image (Fig. 2d), which was well matched with the crystallographic model of Ag_2_MnO_4_ structure (Supplementary Fig. 11). Furthermore, the ABF intensity profile showed the corresponding peaks of Ag_2_MnO_4_ structure (Fig. 2e). We also carried out a series of imaging and analysis for the different zones such as [123], [122], and [311] of the SMNp, and the results were summarized in Supplementary Fig. 12 in the same manner as those presented in Fig. 2. In these projections, the atomically resolved Ag_2_MnO_4_ structure was well observed, as confirmed by crystallographic models. In comparison to this, the electron microscopy and spectroscopy results of the PM showed a typical tetragonal crystal structure (Supplementary Fig. 13).

The detailed elemental composition and distribution of the SMNp was investigated by using STEM–EDX mapping and point spectrum, and this revealed that silver and manganese atoms were evenly distributed in the plate-like structure (Supplementary Figs. 14 and 15). Interestingly, the average atomic ratio of silver and manganese elements on the SMNp was approximately 2, which strongly supported the formation of Ag_2_MnO_4_ structure (Supplementary Table 2). A series of EEL spectra were randomly collected from the STEM image (Fig. 2f), where all sites showed the identical Ag M_4,5_-edge, O K-edge, and Mn L_2,3_-edge (Fig. 2g). This implies that the plate-like region of the SMNp consists of the Ag_2_MnO_4_ structure.

### Electrocatalyst activity and stability

**Fig. 3:**
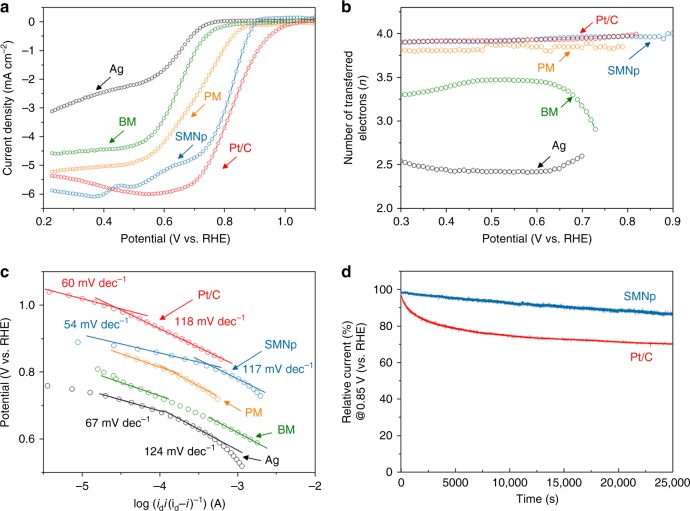
Electrocatalytic activities for oxygen reduction reaction. **a** Linear scan voltammogram (LSV) curves for the Ag nanoparticle, bulk manganese oxides (BM), polyhedron manganese oxide (PM), silver manganate nanoplate (SMNp), and a commercial Pt/C catalyst at a rotating ring–disk electrode (1600 r.p.m) in O_2_-saturated 0.1 M KOH solution with a scan rate of 5 mV s^–1^. All data have been calibrated by using Ar-saturated 0.1 M KOH solution. **b**, **c** The corresponding number of transferred electrons (**b**) and Tafel plots (**c**) of the Ag, BM, PM, SMNp, and Pt/C. I_d_ represents the limiting current density obtained at a potential of 0.30 V in **a**. **d** Chronoamperometric response of the SMNp and 20 wt% Pt/C in O_2_-saturated 0.1 M KOH electrolytes at 0.85 V (vs. RHE) with 1600 r.p.m showing good stability of SMNp compared with Pt/C

To investigate the ORR activity of the SMNp, we measured linear sweep voltammetry (LSV) using a rotating ring–disk electrode (RRDE) in O_2_-saturated electrolyte solution of 0.1 M KOH solution at 24 °C (Fig. 3a). For comparison, the ORR performances of Ag, BM, PM, and commercial 20 wt% Pt/C were also measured under the same condition. The pristine Ag nanoparticle showed the poor catalytic activity with respect to both onset potential and limiting current density. The catalytic activity of the BM and PM was higher than that of Ag nanoparticle, showing positively shifted onset potentials. Although the PM had the highest specific surface area (14.18 m^2^ g^–1^), the ORR activity was limited because it contained the Mn_5_O_8_ phase having low electrical conductivity^55^. For the SMNp, we observed a much higher onset and half-wave potentials of 0.90 ± 0.01 and 0.80 ± 0.01 V vs. RHE (reversible hydrogen electrode) than the other samples, which was little smaller than the onset (1.05 ± 0.01 V vs. RHE) and half-wave potential (0.85 ± 0.01 V vs. RHE) of the Pt/C. It was clear that the existence of silver nanoparticles in the SMNp could significantly decrease the overpotential due to its high electrical conductivity, promoting electron transfer processes. The fluctuating current density of the SMNp at low-voltage range may result from capacitance correction because of large non-faradaic capacitance (Supplementary Fig. 16). The ORR activities of the control samples with different contents of precursors were shown in Supplementary Fig. 17. We found that the SMNp with an optimized content of silver nanoparticle (0.10 g), manganese source (0.07 g), and reducing agent (0.6 mL) showed the best electrochemical performance. We also conducted the rotation speed-dependent polarization curves for the SMNp to estimate the reaction pathways for the ORR (Supplementary Fig. 18). The Koutecky−Levich (K−L) plots of the SMNp showed the nearly parallel linear plots, which was the first-order reaction with respect to oxygen.

To elucidate the reaction pathway for the ORR, we calculated the value of electron transfer number per oxygen molecule (*n*). The number of electrons for the SMNp was ~3.9, which was obviously higher than that for Ag, BM, and PM (Fig. 3b), implying that the direct four-electron pathway was governed during the ORR process^56^. Further, we confirmed the excellent ORR activity of the SMNp from the mass transfer-corrected Tafel plots (Fig. 3c). The slope of the SMNp at a low overpotential region showed the lowest value of 54 mV dec^−1^, which was even lower than Pt/C (60 mV dec^−1^). This result also indicated that the rate-determining step of both the SMNp and Pt/C was the formation of superoxides by one electron transfer^57^. In addition, a larger kinetic current density of the SMNp (1.13 mA cm^−2^ at 0.85 V vs. RHE) indicated a higher ORR activity, in comparison to the Ag nanoparticle, BM, and PM (Supplementary Fig. 19). Such improvements of the SMNp can be explained by the intragranular cracks and abundant dislocations in nanoplate structures, providing a wide range of oxygen adsorption and desorption sites for facile ion transport^53^. In terms of the manganese oxidation state, the mixed valence state (Mn^3+/4+^) of the SMNp confirmed by the XPS measurement could promote the oxygen bond cleavage^40^. In addition to the ORR activity, the electrochemical stability of the catalysts is another important factor, thus, we measured its chronoamperometric (i–t) response for the ORR (Fig. 3d). Interestingly, the durability of the SMNp showed ~17.4% higher than that of the Pt/C, which could be ascribed to ~200 mV higher equilibrium potential of Ag/Ag_2_O than that of Pt/PtO in alkaline solution^34^.

### Aluminum–air flow battery performance

**Fig. 4:**
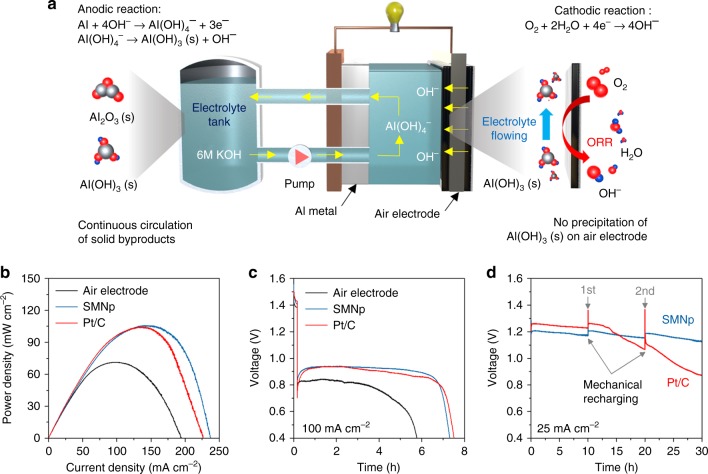
Electrochemical performance of aluminum–air flow batteries. **a** Schematic of the aluminum–air flow battery (AAFB) system, which includes a single stack cell, one electrolyte tank, and circulation pump. ORR indicates oxygen reduction reaction. **b** Power density curves of flow cells using the pristine air electrode, silver manganate nanoplate (SMNp), and Pt/C with 6 M KOH electrolyte (scan rate of 0.1 mA s^−1^). **c** Discharge curves using the pristine air electrode, SMNp, and Pt/C at 100 mA cm^−2^. **d** Mechanical charge and discharge tests using the SMNp and Pt/C at 25 mA cm^−2^, where the aluminum and electrolyte were replaced every cycle

The electrochemical performance of the SMNp catalyst was evaluated by aluminum–air flow batteries (AAFBs). As shown in Fig. 4a, we designed a flow-type aluminum–air cell, and this cell assembly consists of an aluminum metal anode, catalyst-loaded air electrode, and alkaline electrolyte tank (Supplementary Fig. 20). By supplying fresh electrolyte, we can avoid the precipitation of by-products, making the surface of electrodes clean^2^. In addition, self-discharge problem can be prevented by draining off the electrolyte, ensuring a long shelf life of the AAFBs. In comparison to a conventional static cell, the AAFB demonstrated improved peak power density of ~71.2 mW cm^−2^ at 100 mA cm^−2^, and the discharge duration of the AAFBs was 11 times longer than the static cell at 50 mA cm^−2^ (Supplementary Fig. 21a, b). In terms of the by-products precipitation, the surface of aluminum anode in a static cell was covered with a solid Al(OH)_3_ precipitate, thus causing severe cell degradation with low discharge capacity (Supplementary Fig. 21c, d).

To further increase the battery performance, we used catalysts loaded with air electrodes (Ag nanoparticle, BM, PM, SMNp, and Pt/C) in the AAFBs. The discharge process was initiated after 10 min of rest with a circulating electrolyte to activate aluminum anode by eliminating the surface oxide phase. As shown in Fig. 4b and Supplementary Fig. 22, the SMNp demonstrated significantly enhanced peak power density of ~105.2 mW cm^−2^_,_ compared to the pristine air electrode. This value was comparable to that of the Pt/C (~104.0 mW cm^−2^), and the maximum current density of the SMNp (~237 mA cm^−2^) was even higher than that of the Pt/C (~226 mA cm^−2^). Power density results of other samples were summarized in Supplementary Table 3. The effects of the ZnO additive in 6 M KOH electrolytes are shown in Supplementary Fig. 23, which increases the discharge voltage of ~0.11 V. However, we conducted flow cell tests without any additives to clearly demonstrate the electrolyte flowing system. The galvanostatic discharge tests were conducted at different constant currents of 50 and 100 mA cm^−2^. The specific capacity of the SMNp was ~2642 A h kg_Al_^−1^ at 50 mA cm^−2^, which was calculated on the basis of aluminum weight (Supplementary Fig. 24). When the current density increased to 100 mA cm^−2^, the SMNp showed discharge time of 7.3 h corresponding to the specific capacity of ~2843 A h kg_Al_^−1^, approaching the theoretical specific capacity of 2978 A h kg_Al_^−1^ (Fig. 4c and see Supplementary Note 1). Notably, the gravimetric and volumetric energy densities of the SMNp were ~2552 W h kg_Al_^−1^ and ~6890 W h L_Al_^−1^ at 100 mA cm^−2^, and these values were comparable with those of the Pt/C (~2507 W h kg_Al_^−1^ and ~6796 W h L_Al_^−1^), which was summarized in Supplementary Table 4. When the current density increased to 100 mA cm^−2^, the open-circuit potentials of the air electrode, SMNp, and Pt/C were ~1.42, ~1.55, and ~1.50 V, which were decreased after initiating the discharge process because of high current density (Fig. 4c). When the cells were subjected to the constant voltage test at 1 V (Supplementary Fig. 25), the SMNp exhibited the maximum current density of ~87 mA cm^−2^, which was higher than that of the Pt/C (~82 mA cm^−2^). This outstanding electrochemical performance could be originated from the synergistic effects of silver nanoparticle with high electrical conductivity and plate-like, striped Ag_2_MnO_4_ structure with abundant dislocations.

Remarkably, the AAFB with the SMNp showed excellent cycle stability upon a mechanically charging process, where the electrolyte and aluminum plate were replaced after fully discharging at 25 mA cm^−2^ (Fig. 4d). At the initial stage, the Pt/C showed relatively lower overpotentials than the SMNp, showing a discharge potential of ~1.24 V. However, from the second cycle, the significant potential drop was observed in the Pt/C because of degradation of platinum element in concentrated alkaline solution. The SMNp presented stable discharge curves during prolonged cycles, suggesting its chemical stability, which was agreed with the chronoamperometric response in Fig. 3d. We also confirmed that the SMNp catalyst was stably maintained after battery cycling by SEM–EDX analysis (Supplementary Fig. 26). Therefore, the choice of the silver-based materials offers the large potentials of developing high-energy AAFBs with a minimal cell degradation.

## Discussion

**Fig. 5:**
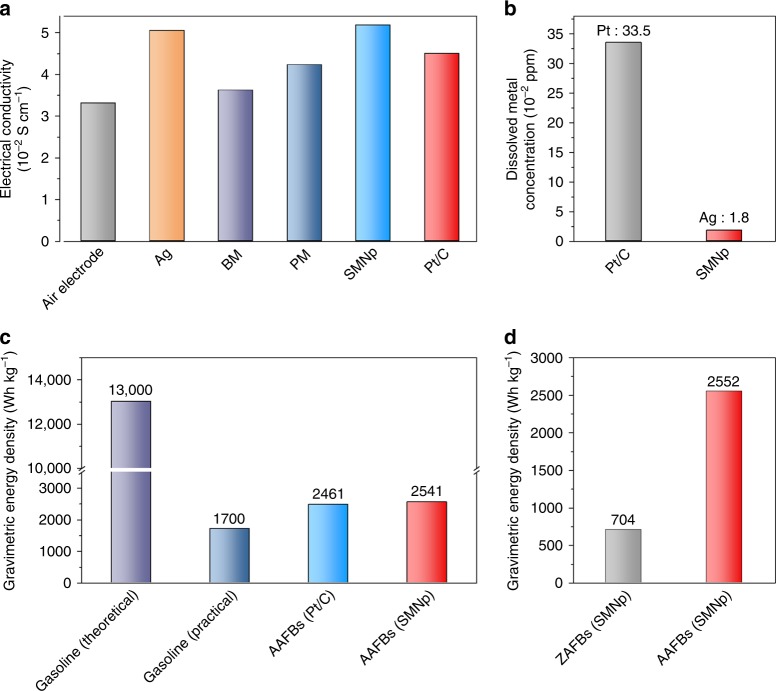
Comparison of electrode properties and battery performance. **a** Electrical conductivity of the Ag nanoparticle, bulk manganese oxides (BM), polyhedron manganese oxide (PM), silver manganate nanoplate (SMNp), and Pt/C-loaded air electrodes. **b** Precious metal dissolution tests in aluminum–air flow batteries (AAFBs) using the SMNp and Pt/C with 6 M KOH electrolyte after 6 h of discharging at 50 mA cm^−2^. **c**, **d** Comparison of the gravimetric energy density (**c**) among gasoline with theoretical and practical value, AAFBs with Pt/C and SMNp (at 50 mA cm^−2^), and comparison of the gravimetric energy density (**d**) between zinc–air flow batteries (ZAFBs) and AAFBs with the SMNp at 100 mA cm^−2^

The origin of the improvement of cell potential and stability by the SMNp catalyst can be understood with respect to the electrical conductivity of the electrode and the degree of metal dissolution. First, we measured electrical conductivity of the catalyst-loaded air electrodes by using a through-plane conductivity measurement setup (Supplementary Fig. 27)^58^. As shown in Fig. 5a, the electrical conductivity of the Ag-loaded air electrode (5.05 × 10^−2^ S cm^−1^) was higher than that of the Pt/C-loaded one (4.01 × 10^−2^ S cm^−1^) because of lower electrical resistivity of silver than platinum. Interestingly, the SMNp revealed the highest electrical conductivities of 5.18 × 10^−2^ S cm^−1^, which could be ascribed to the silver nanoparticle-distributed nanoplate configuration. This also implied that the intrinsic drawback of dislocation having poor grain-to-grain connection can be overcome by the silver element^53^. The electrical properties of all samples are summarized in Supplementary Table 5.

At a current density of < 100 mA cm^−2^, we confirmed that the discharge voltage of the Pt/C was substantially higher than that of the SMNp in polarization curves (Supplementary Fig. 22), which was well matched with the results from LSV curves (Fig. 3a). However, the discharge voltage of the SMNp has started to be comparable with that of the Pt/C at a current density of > 100 mA cm^−2^, which eventually exceeded the discharge voltage of Pt/C. These phenomena were also confirmed by the individual discharge curves of the SMNp and Pt/C with different current densities of 25 (Fig. 4d), 50 (Supplementary Fig. 24), and 100 mA cm^−2^ (Fig. 4c). When we carefully observed the discharge curves in relatively low current densities of 25 and 50 mA cm^−2^, the overpotentials for the SMNp were higher than Pt/C, thus lowering the discharge voltage. However, the SMNp showed a comparable voltage profile to Pt/C at a high current density of 100 mA cm^−2^, which could be ascribed to the superior electrical conductivity of the SMNp. This was confirmed by measuring the electrical conductivity of catalysts-loaded air electrode itself (Fig. 5a and Supplementary Fig. 27). Therefore, under the current density of > 100 mA cm^−2^, the electrical conductivity of the electrode plays a key role for enhancing the overall electrochemical performance of aluminum–air flow battery.

In terms of the chemical stability, we analyzed the dissolution of precious metal after cycling by using inductively coupled plasma mass spectrometry (ICP-MS). The electrolytes were collected after the AAFB was discharged for 6 h at 50 mA cm^−2^ (Fig. 5b). The cell equipped with the Pt/C showed a concentration of platinum dissolution of 33.5 × 10^−2^ ppm. For the SMNp, we observed that the concentration of silver dissolution was 18 times lower than that of platinum (1.8 × 10^−2^ ppm). This exceptional chemical stability of silver element in the SMNp can reasonably support the results of chronoamperometric response and mechanical recharging test.

We compared the gravimetric energy density of our AAFB with gasoline^59^ (Fig. 5c). It was reported that the theoretical energy density of gasoline was ~13,000 W h kg^−1^ (~9500 W h L^−1^). However, the practical energy density of gasoline is ~1700 W h kg^−1^ (~1200 W h L^−1^) considering the average tank-to-wheel efficiency of 12.6%.^60^ Compared to this, the gravimetric energy density of our AAFBs with SMNp was ~2552 W h kg_Al_^−1^, which showed large potentials for the aluminum as an alternative energy source. The volumetric energy density of each system was also summarized in Supplementary Fig. 28. Finally, we showed that the SMNp catalyst can be also used in zinc–air flow batteries (ZAFBs) and compared with AAFBs (Fig. 5d, Supplementary Fig. 29 and Table 6). The ZAFBs with SMNp showed the discharge capacity of ~558 mA h and gravimetric energy density of ~704 W h kg_Zn_^−1^ at a current density of 100 mA cm^−2^, which still lags behind the performance of the AAFBs. Notably, the amount of consumed metal per discharge capacity in the AAFBs was ~0.35 g A h^−1^, in comparison to the value of ~1.24 g A h^−1^ in the ZAFBs, indicating three times higher conversion efficiency of the AAFBs.

In conclusion, we have prepared a silver nanoparticle seed-mediated silver manganate nanoplate architecture for ORR. We discovered that the silver atom can migrate into the available crystal lattice and rearrange manganese oxide structure, thus creating abundant surface dislocations. The activity and durability of this material outperformed the platinum-based electrocatalyst in concentrated alkaline solution. Furthermore, we have shown that flow-based aluminum–air battery with our catalyst not only offers a route to stable, robust energy conversion device, but also brings unprecedented gravimetric and volumetric energy densities of ~2507 W h kg_Al_^−1^ and ~6890 W h L_Al_^−1^, as summarized in Supplementary Table 7. This innovative strategy prevented the precipitation of solid by-product in the cell and dissolution of a precious metal in air electrode. We believe that our AAFB system has the potential for a cost-effective and safe next-generation energy conversion system.

## Methods

### Catalyst synthesis

The silver nanoparticle-mediated silver manganate nanoplate (SMNp) was synthesized by the simple precipitation and heat-treatment process. First, 0.07 g of manganese(II) nitrate hydrate (≥98%, Sigma-Aldrich) and 0.1 g of silver nanoparticle (polyvinylpyrrolidone functionalized, particle size of <100 nm, ≥99.5%, Sigma-Aldrich) were subsequently added into 2 mL of ethanol solution under vigorous stirring with a magnetic bar. Next, to form a uniform coating manganese layer on silver nanoparticles, 0.6 mL of 8 M NaOH solution as a reducing agent was prepared by sodium hydroxide beads (≥98%, SAMCHUN), and then poured into the above-blended solution. After finishing the reduction of manganese ions and coating process, the solution was centrifuged at 4000 r.p.m. for 5 min with DI water and dried in a 200 °C oven for overnight. The resultant composite was annealed at 450 °C for 15 min at a heating rate of 5 °C min^−1^ under ambient air atmosphere to form silver manganate nanoplate structure. The control experiments with different contents of silver nanoparticles (0.05 and 0.2 g), manganese(II) nitrate hydrate (0.035 and 0.140 g), and 8 M NaOH solution (0.15 and 2.40 mL) were conducted with the same procedure above.

### Structural characterization

The morphology of the synthesized materials was examined using scanning electron microscopy (SEM, VERIOS 460, FEI) operating at 10 kV and high-resolution transmission electron microscope (HR-TEM, ARM300, JEOL) operating at 300 kV. Elemental mapping was attained using energy-dispersive X-ray spectroscopy (EDX) equipped with the SEM and TEM. The crystal structure analysis was performed using an X-ray diffractometer (XRD, D/Max2000, Rigaku) with Cu-Ka radiation. Surface chemistry was analyzed using X-ray photoelectron spectroscopy (XPS, Thermo Scientific Kα spectrometer, 1486.6 eV)

### Preparation of catalyst inks and working electrode

The catalyst ink was prepared using a mixture of 16 mg of the catalyst powder, 4 mg of a Ketjenblack, 800 µL of ethanol, and 200 µL of 5 wt% Nafion solution (Sigma-Aldrich). The Pt/C catalyst ink was made by adding 10 mg of Pt/C based on Vulcan XC 72 (20 wt%, Premetek Co.) powder into 800 µL of ethanol and 200 µL of 5 wt% Nafion solution. The catalyst inks were homogeneously mixed by ultrasonication, and then, 5 µL of the ink was loaded on the surface of the polished glassy carbon of the rotation ring–disk electrode (RRDE) as a working electrode. After loading ink, the working electrode was dehydrated by the vacuum chamber for 30 min. As a result, the loading levels of the synthesized catalysts and Pt/C were 0.637 mg_cat_ cm^–2^ and 0.398 mg_(20% Pt/C)_ cm^–2^, respectively (79.6 μg_pt_ cm^–2^ for the pure Pt amount).

### Electrochemical measurements

The rotating ring–disk electrode (RRDE, ALS Co., Ltd.) measurement was conducted using the catalyst films that were deposited on a glassy carbon electrode in 0.1 M KOH electrolyte saturated with oxygen at 1600 r.p.m. A Pt wire and Hg/HgO were used as a counter and a reference electrode, respectively. Electrochemical characterization was carried out using a bipotentiostat (IviumStat) with ohmic drop correction. The ORR current was measured after the current had saturated by using a cyclic voltammetry (CV) measurement that was made for voltages of 0.20 to –0.67 V (vs. Hg/HgO) at a scan rate of 10 mV s^–1^. The capacitive current was corrected by means of a CV measurement in 0.1 M KOH electrolyte saturated with argon. The measured potentials (vs. Hg/HgO) were calibrated with respect to a reversible hydrogen electrode (RHE) value by adding 0.929 V to the measured potential (vs. Hg/HgO). The number of transferred electrons (*n*) was calculated using the equation below. The number of transferred electrons (*n*) was calculated using the equation below where the collection efficiency (*N*) was determined using 10 mM K_3_[Fe(CN)_6_] electrolyte under argon atmosphere, which was around 0.41. This value is similar to the theoretical value of 0.42. *Id* and *Ir* represent disk and ring currents, respectively.

$${n} = 4\frac{{I_d}}{{I_d + I_r{\mathrm{/}}N}}$$

The electrochemical properties of the AAFBs were evaluated by using a potentionstat/galvanostat (WBC3000, WonAtech) with a voltage cutoff of 0.4 V for a primary flow cell test and 10 h of discharge per cycle for a mechanically charging process, respectively.

### Kinetic current calculation

Kinetic current of electrocatalysts was obtained from mass transfer correction using Levich equation.

$$i_{\mathrm{measured}}^{-1} = i_{k}^{-1} + i_{d}^{-1}$$

where *i*_measured_ is the measured oxygen reduction current density, *i*_*k*_ is the kinetic current density, and i_*d*_ is the limiting current density.

### Aluminum–air flow cell tests

For aluminum–air flow cell test based on the SMNp catalyst, a flow cell kit was fabricated in laboratory scale. Aluminum plate (alloy 6061, 97.93%, Alfa-Aesar) was used as an anode and 40 ml of 6 M KOH as an electrolyte. No separator was used and the air electrode was prepared via a homogenizer to form homogeneous gas diffusion layer (GDL). First, activated charcoal (Darco G60A, Sigma-Aldrich) and catalysts (including Pt/C) were mixed without solvents at 5000 r.p.m. for 2 min. Next, the slurry with the same loading levels for all catalysts was fabricated from a dry-mixed activated charcoal/catalyst mixture and polytetrafluoroethylene binder (60 wt% PTFE emulsion in water, Sigma-Aldrich) at a weight ratio of 6:1:3 with 4 ml of isopropanol and 2 ml of acetone at 5000 r.p.m. for 30 min and pasted on the nickel foam using roll press machine with a thickness of ~700 μm. The pristine air electrode was made by the same procedure at a weight ratio of 7:3 for activated charcoal and PTFE binder. The GDL was hot-pressed at 120 °C for 5 min attached with a PTFE film to ensure proper gas distribution and avoid leakage problems. For a mechanically rechargeable test, the aluminum plate and electrolyte were replaced after discharging at 25 mA cm^−2^ for 10 h. The air electrodes were continuously used without replacement to confirm the cycle stability. The flow rate of electrolytes for flow cell test was 100 mL min^−1^.

## Electronic supplementary material

Supplementary Information

Peer Review File

## Data Availability

The data that support the findings of this study are available from the corresponding author upon request.
