# Supplementary material for: Seed-mediated atomic-scale reconstruction of silver manganate nanoplates for oxygen reduction towards high-energy aluminum-air flow batteries
Source: Nat Commun. 2018 Sep 13;9:3715. doi: 10.1038/s41467-018-06211-3 (PMC6137061; doi:10.1038/s41467-018-06211-3)
Supplement: Supplementary file 1 — Supplementary Information [file 41467_2018_6211_MOESM1_ESM.pdf]

Supplementary Information

Seed-mediated atomic-scale reconstruction of silver manganate nanoplates for oxygen reduction towards high-energy aluminum-air flow batteries

Ryu et al.

Supplementary Note 1. Calculations of theoretical energy densities for aluminum-air batteries.

Cathode : $O_{2(g)} + 2H_2O_{(l)} + 4e^- \rightarrow 4OH^-_{(aq)}$ (E^o = 0.4 V vs. NHE)

Anode : $Al_{(s)} + 4OH^-_{(aq)} \rightarrow Al(OH)_4^-{}_{(aq)} + 3e^-$ (E^o = − 2.33 V vs. NHE)

$Al(OH)_4^-{}_{(aq)} \rightarrow Al(OH)_{3(s)} + OH^-_{(aq)}$

$Al_{(s)} + 3H_2O_{(l)} + OH^-_{(aq)} \rightarrow Al(OH)_4^-{}_{(aq)} + 3/2H_{2(g)}\uparrow$ (also possible)

Overall : $Al_{(s)} + 3/4O_{2(g)} + 3/2H_2O_{(l)} \rightarrow Al(OH)_{3(s)}$ (E^o = 2.73 V vs. NHE)

Molar mass of the reactant → Al = 27 g/mol, O_2 = 32 g/mol, H_2O = 18 g/mol

Calculation of the gravimetric energy density

$$\frac{3\ mol\ e^-}{1\ mol\ Al}\times\frac{96500\ A\ s}{1mol\ e^-}\times\frac{1\ mol\ Al}{78\ g}\times\frac{10^3\ g}{1\ kg}\times\frac{1\ h}{3600\ s}\times 2.73\ V \qquad (1)$$

$$= 2815\ Wh\ kg^{-1}\ (including\ Al, O_2, H_2O)$$

$$\frac{3\ mol\ e^-}{1\ mol\ Al}\times\frac{96500\ A\ s}{1mol\ e^-}\times\frac{1\ mol\ Al}{54\ g}\times\frac{10^3\ g}{1\ kg}\times\frac{1\ h}{3600\ s}\times 2.73\ V \qquad (2)$$

$$= 4066\ Wh\ kg^{-1}\ (including\ Al, H_2O)$$

$$\frac{3\ mol\ e^-}{1\ mol\ Al}\times\frac{96500\ A\ s}{1mol\ e^-}\times\frac{1\ mol\ Al}{27\ g}\times\frac{10^3\ g}{1\ kg}\times\frac{1\ h}{3600\ s}\times 2.73\ V \qquad (3)$$

$$= 8131\ Wh\ kg^{-1}\ (including\ Al\ only)$$

Molar volume of the reactant → Al = 27 g/mol ÷ 2700 g/L = 1×10^{-2} L/mol

→O_2 = 32 g/mol ÷ 1.43 g/L = 22 L/mol

→H_2O = 18 g/mol ÷ 1000 g/L = 1.8×10^{-2} L/mol

Calculation of the volumetric energy density

$$\frac{3\ mol\ e^-}{1\ mol\ Al} \times \frac{96500\ A\ s}{1 mol\ e^-} \times \frac{1\ mol\ Al}{16.5\ L} \times \frac{1\ h}{3600\ s}\ \times 2.73\ V \tag{4}$$

$$= 13\ Wh\ L^{-1}\ (including\ Al, O_2, H_2O)$$

$$\frac{3\ mol\ e^-}{1\ mol\ Al} \times \frac{96500\ A\ s}{1 mol\ e^-} \times \frac{1\ mol\ Al}{3.7 \times 10^{-2}\ L} \times \frac{1\ h}{3600\ s}\ \times 2.73\ V \tag{5}$$

$$= 5933\ Wh\ L^{-1}\ (including\ Al, H_2O)$$

$$\frac{3\ mol\ e^-}{1\ mol\ Al} \times \frac{96500\ A\ s}{1 mol\ e^-} \times \frac{1\ mol\ Al}{10^{-2}\ L} \times \frac{1\ h}{3600\ s}\ \times 2.73\ V \tag{6}$$

$$= 21954\ Wh\ L^{-1}\ (including\ Al\ only)$$

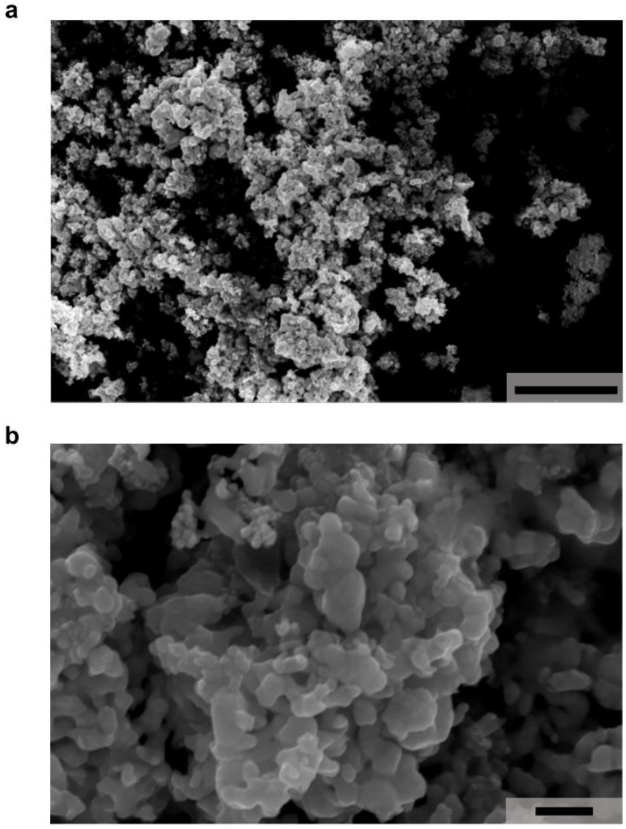

Supplementary Figure 1 | a,**b**, SEM of the Ag (**a**) and its magnified images (**b**). Scale bars, 3 μm (**a**), and 300 nm (**b**).

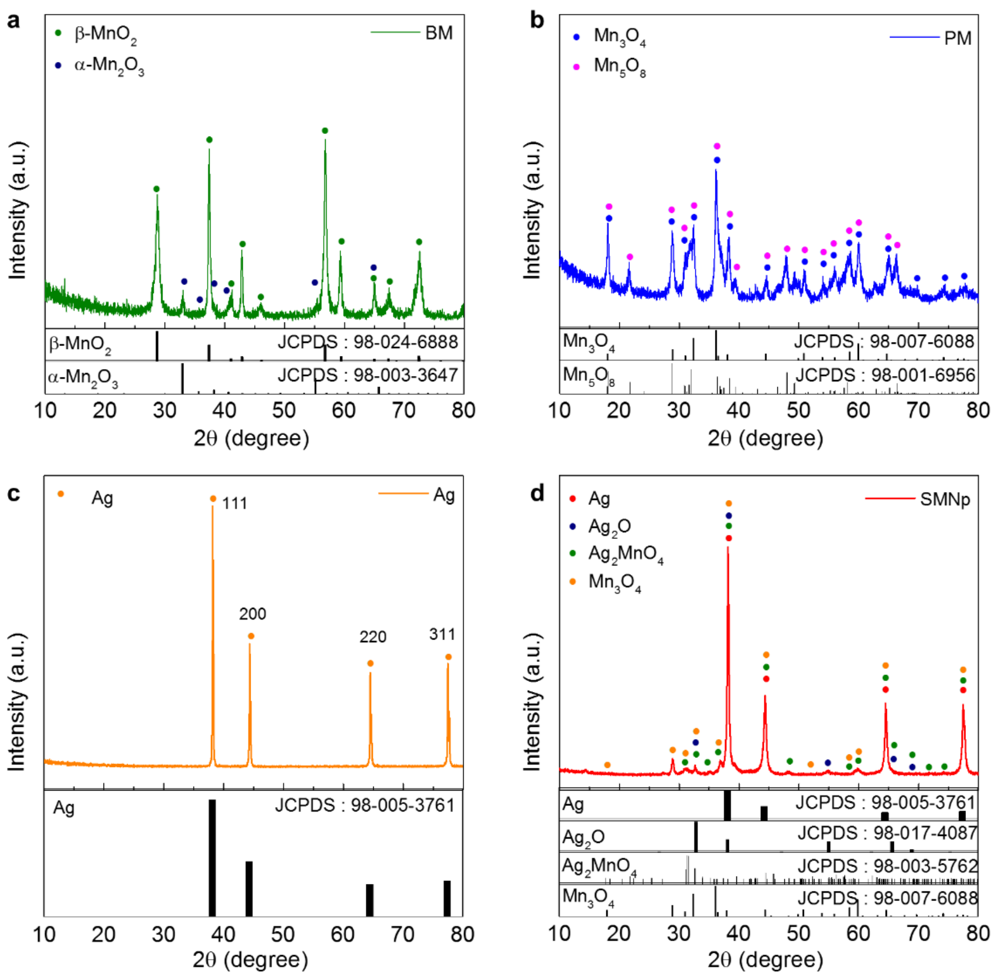

Supplementary Figure 2 | a–**d**, XRD survey of the BM (**a**), PM (**b**), Ag (**c**) and SMNp (**d**) with their JCPDS data.

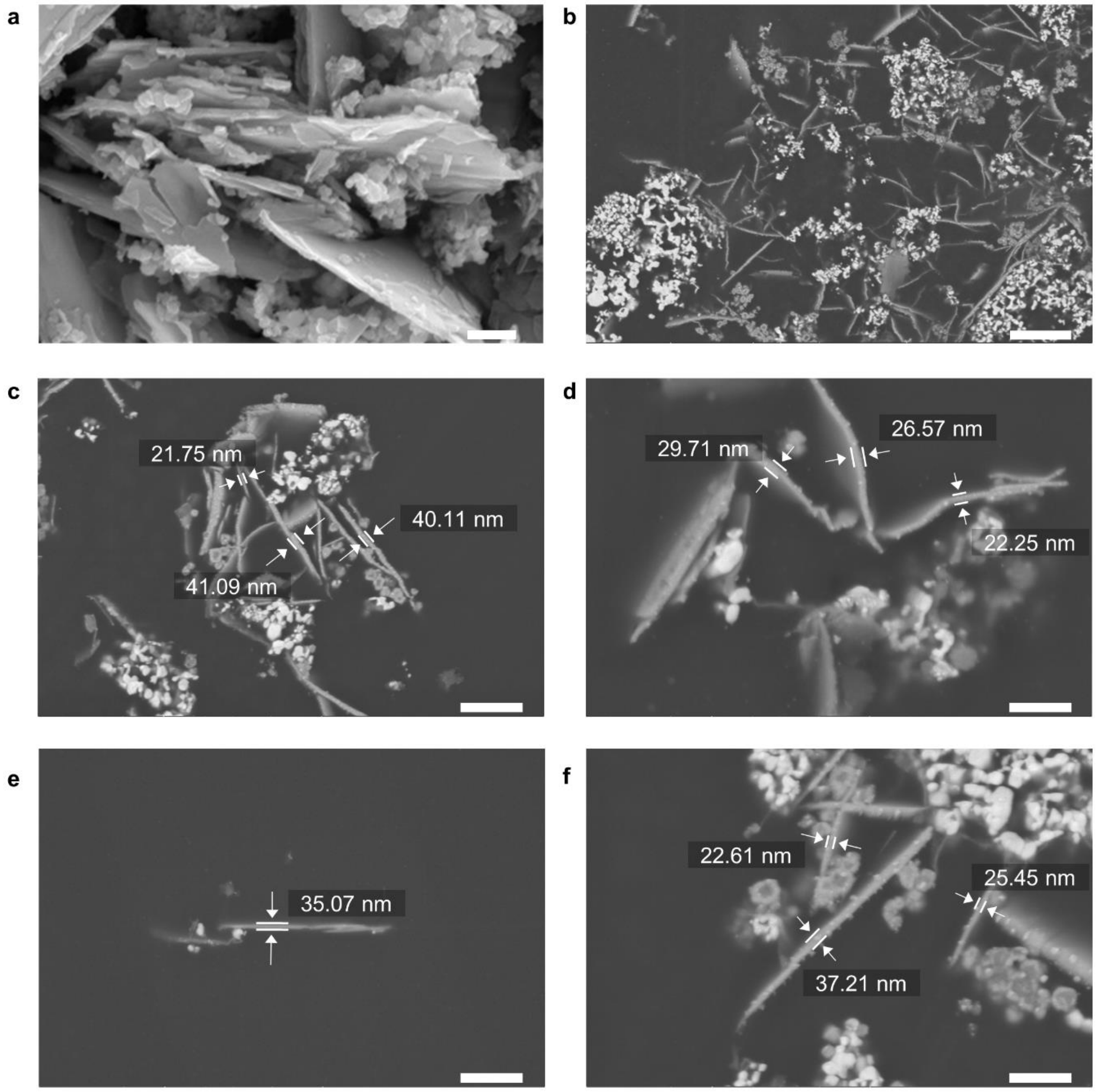

Supplementary Figure 3 | a, SEM image of the SMNp before ion-milling. **b–f**, SEM images of the SMNp after ion-milling, showing an average thickness of ~30.2 ± 0.5 nm, where magnified four SEM images from different regions were included to clearly provide the information about thickness of thinned SMNp sample. Scale bars, 250 nm (**a,d,f**), 1 μm (**b**), and 500 nm (**c,e**).

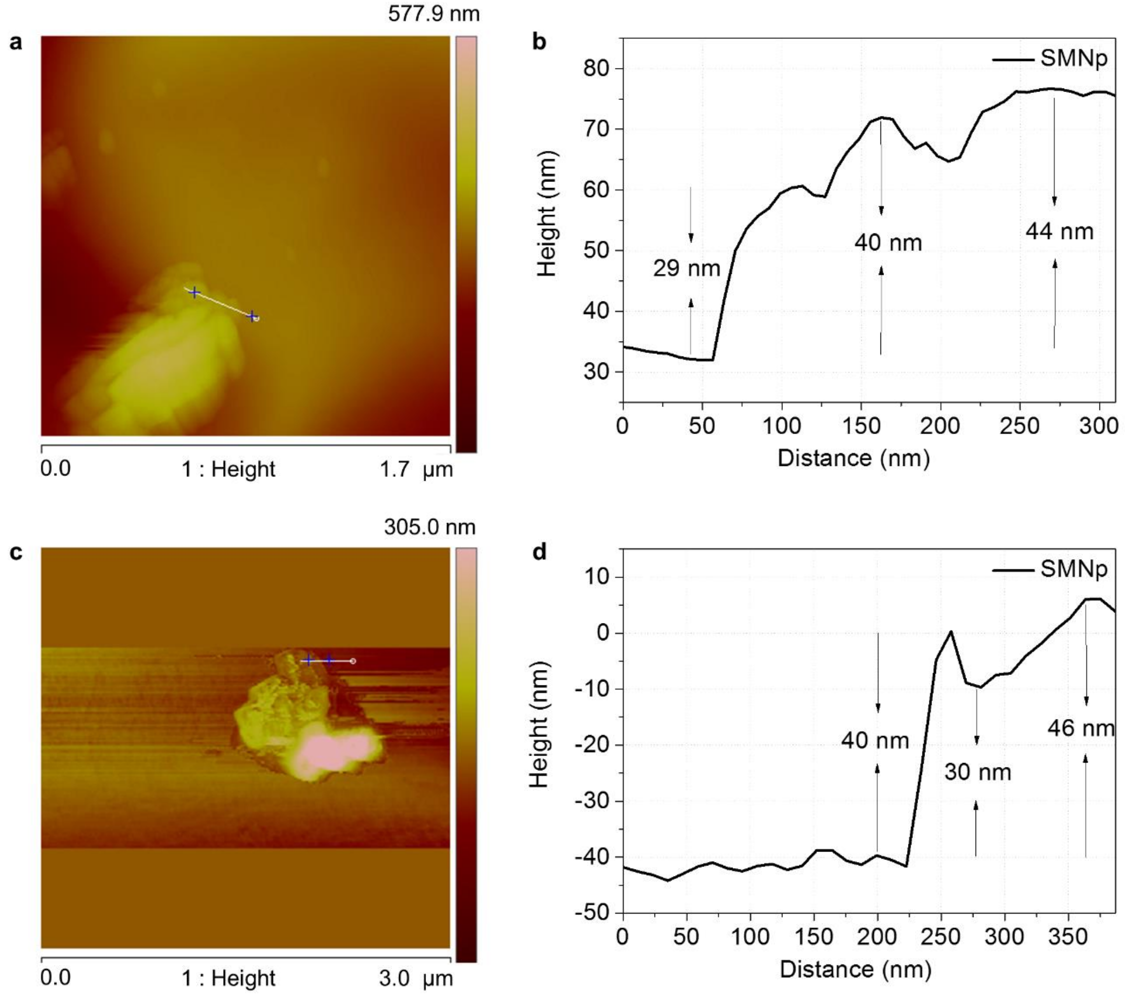

Supplementary Figure 4 | a–d, AFM analysis of the SMNp, showing an average thickness of ~38 nm, where surface of the SMNp are rough due to composite of silver nanoparticles and silver manganate nanoplates.

.

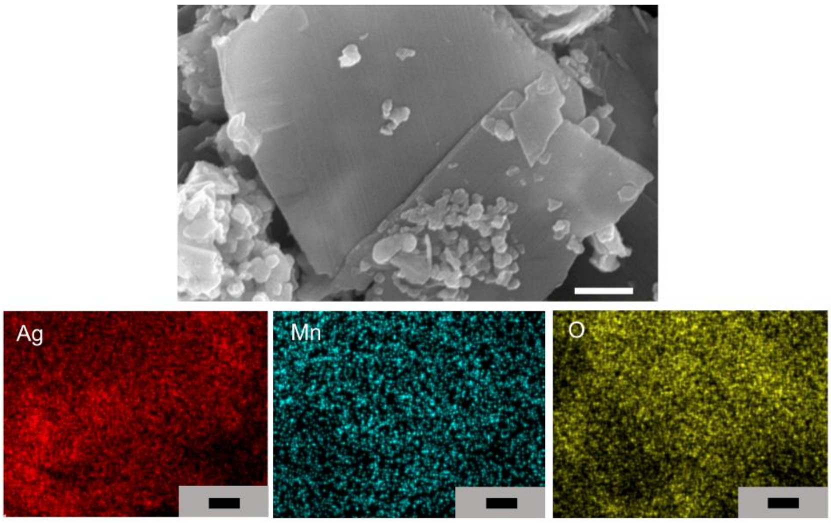

Supplementary Figure 5 | SEM-EDX analysis of the SMNp, showing silver, manganese and oxygen elements. Scale bars, 250 nm.

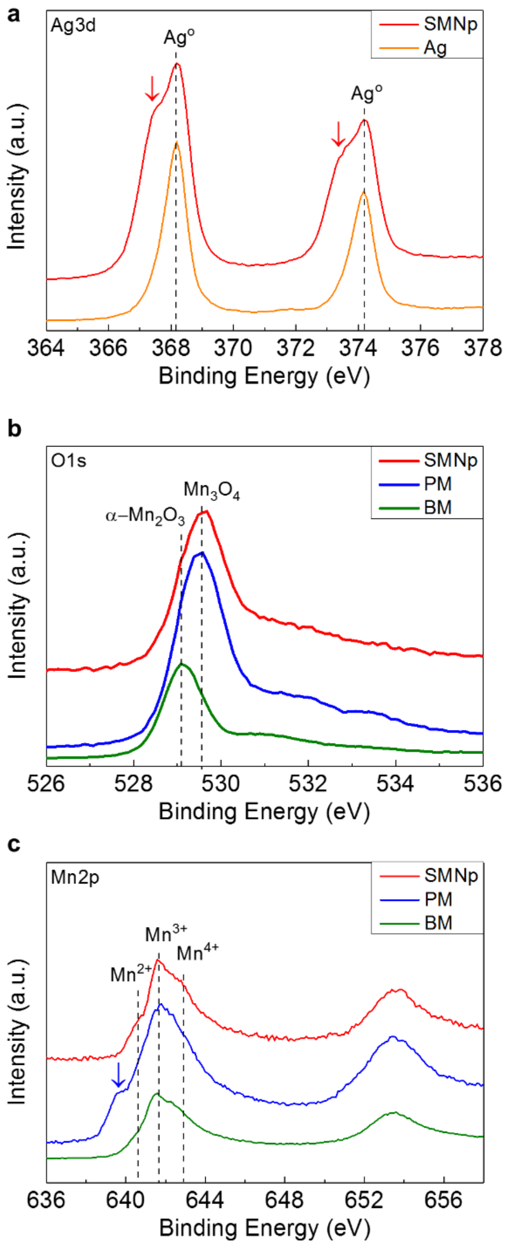

Supplementary Figure 6 | a–c, XPS survey for the Ag, BM, PM and SMNp, representing Ag 3d (**a**), O 1s (**b**), and Mn 2p (**c**) spectra. The arrows in **a** and **c** indicate the formation of Ag_2O and Mn_5O_8, respectively.

Supplementary Table 1 | Specific surface area of the BM, PM and SMNp.

Sample	BM	PM	SMNp
BET surface area ($m^{-2}\ g^{-1}$)	4.97	14.18	11.47

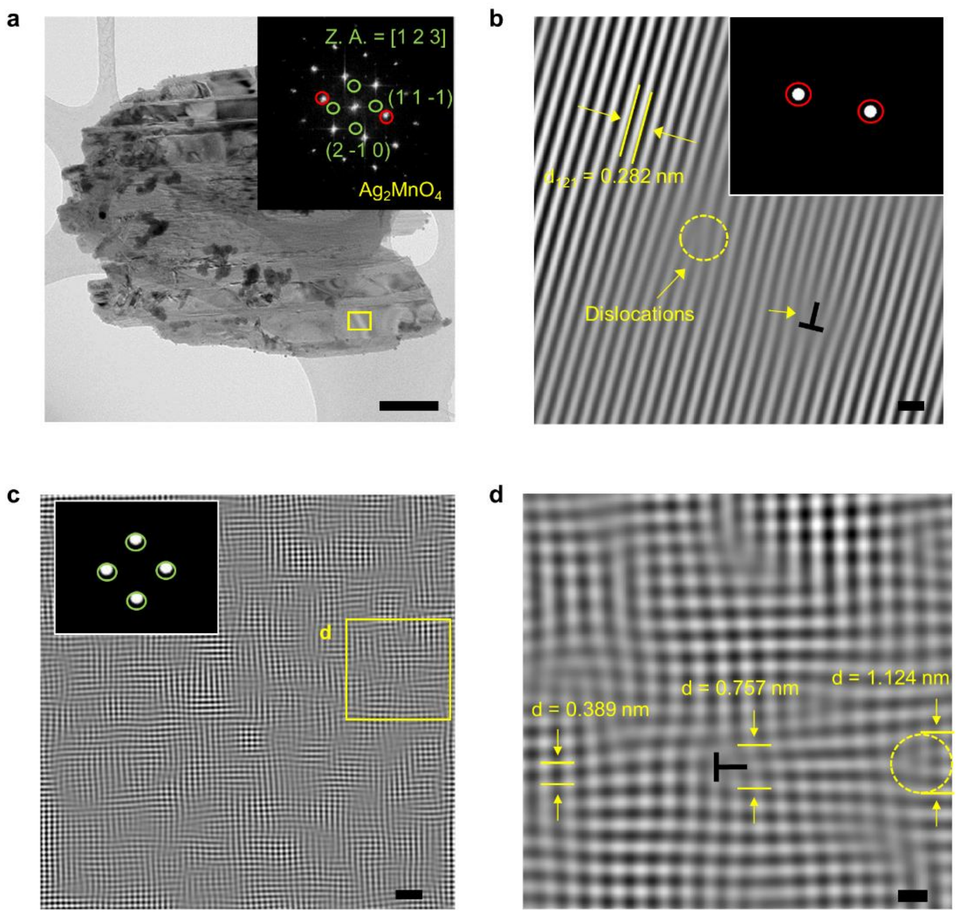

Supplementary Figure 7 | Electron microscopy and spectroscopy of SMNp. a, HR-TEM image of the SMNp with a FFT pattern from yellow box, indicating the lattice planes of (11–1) and (2–10) along the [123] zone axis, presenting orthorhombic structures of Ag_2MnO_4. **b**–**d**, Inverse FFT patterns of applied masks from red (**b**) and green (**c**) circles in **a**, showing a wide range of dislocations with abundant defects, and magnified FFT pattern (**d**) from **c**, showing gradual increase of d-spacing. The dislocations are shown by circles, and edge dislocations are represented by T-shaped symbols. Scale bars, 250 nm (**a**), 0.5 nm (**b,d**), and 2 nm (**c**).

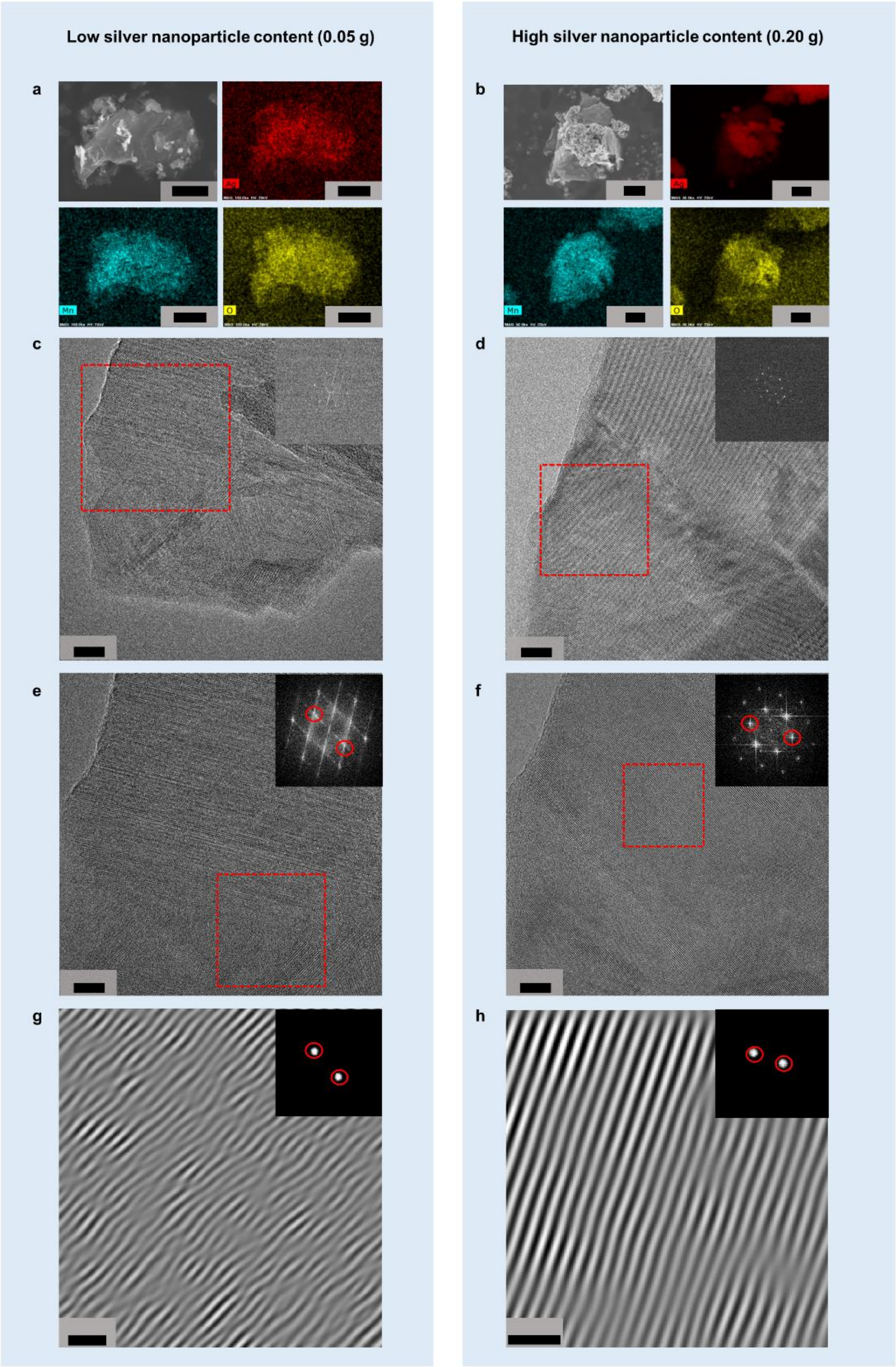

Supplementary Figure 8 | a–d, SEM-EDX mapping image of the SMNp with low (**a**) and high (**b**) amounts of silver nanoparticles, showing successful synthesis of the silver manganate nanoplate, and their HR-TEM images (**c**,**d**) with the SAED patterns at each inset indicating higher crystallinity of **d** than **c**. **e**,**f**, Magnified HR-TEM images of red box in **c** and

d with reduced FFT patterns of red box in **e** and **f**. **g**, Inverse FFT images of applied masks at red circle in **e**, showing excess of dislocations with defects. **h**, Inverse FFT images of applied masks at red circle in **f**, showing clean atomic arrangements with little defects. Scale bars, 400 nm (**a**), 500 nm (**b**), 10 nm (**c**,**d**), 5 nm (**e**,**f**), and 1 nm (**g**,**h**).

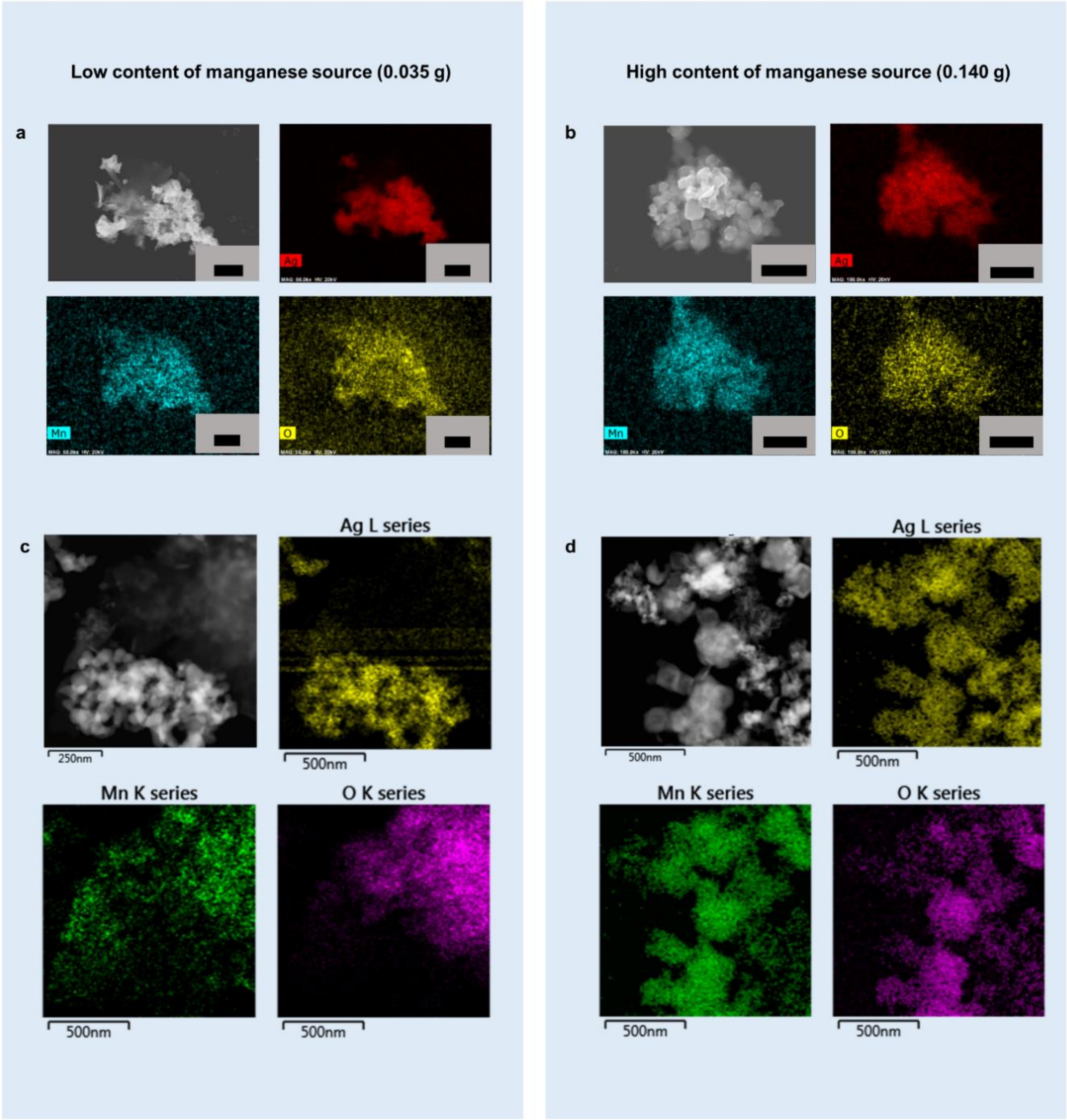

Supplementary Figure 9 | a–**d**, SEM and TEM-EDX images of the SMNp with low manganese (**a**,**c**) and high manganeses amounts (**b**,**d**), showing that nanoplate was not formed with aggregated morphologies. Scale bars, 500 nm (**a**), and 400 nm (**b**).

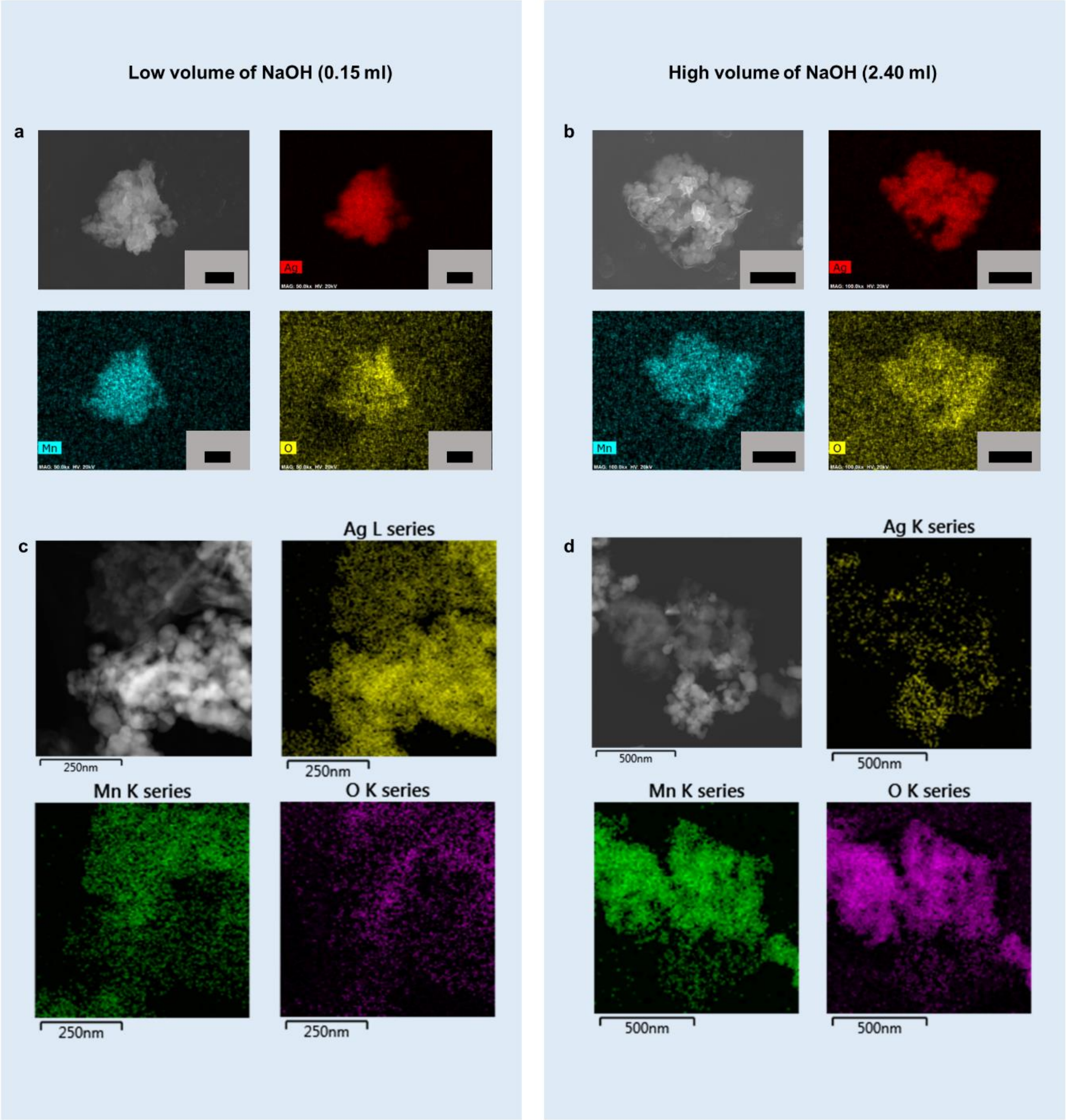

Supplementary Figure 10 | a–d, SEM and TEM-EDX images of the SMNp with low NaOH (**a,c**) and high NaOH volumes (**b,d**), indicating that nanoplate was not formed with aggregated morphologies. Scale bars, 500 nm (**a**), and 400 nm (**b**).

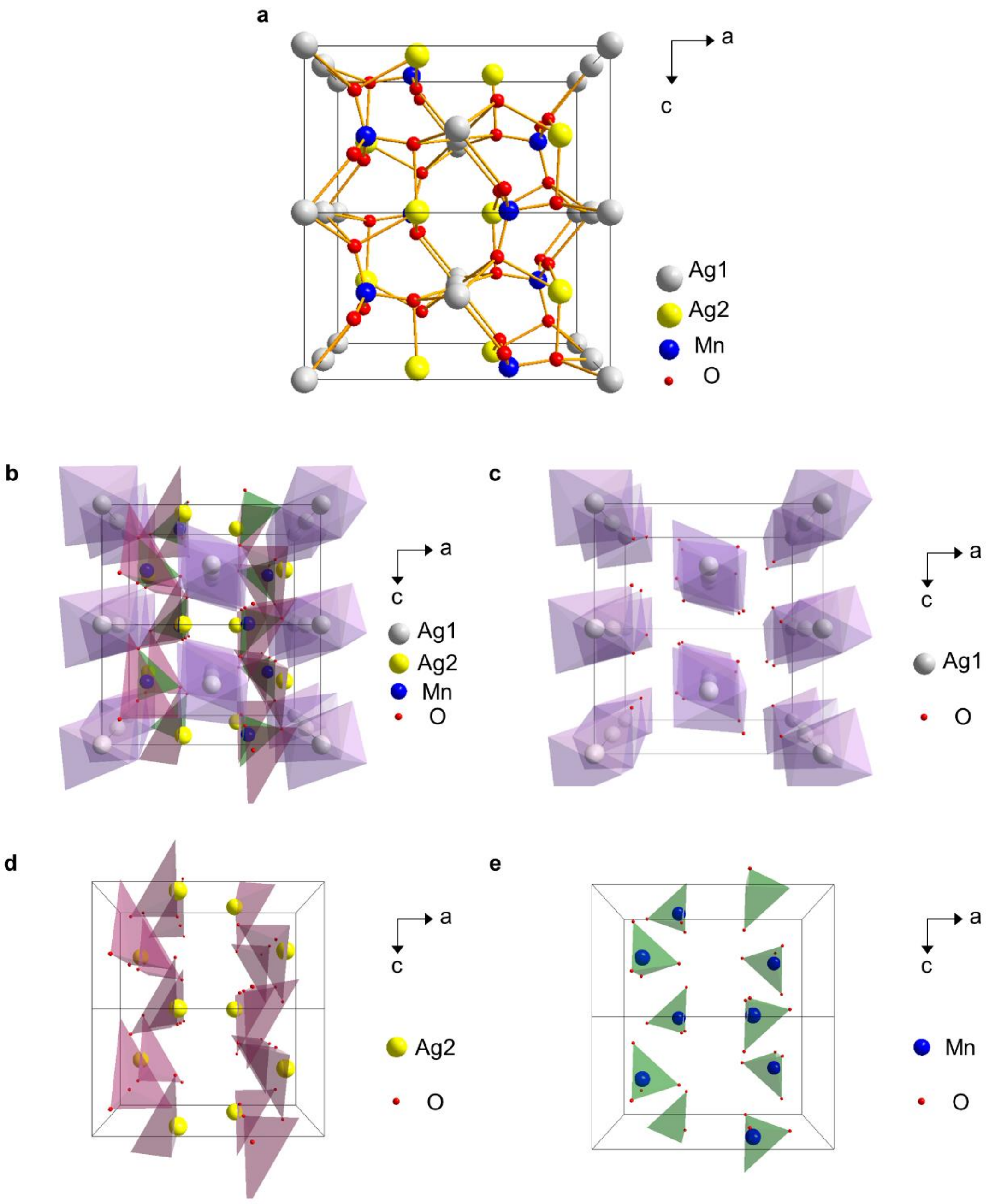

Supplementary Figure 11 | a–e, Crystallographic model and oxygen environments of Ag_2MnO_4 along the [010] zone axis (**a**), presenting complex zig-zag arrangements of oxygen atoms with all elements (**b**), Ag1 (**c**), Ag2 (**d**) and Mn (**e**).

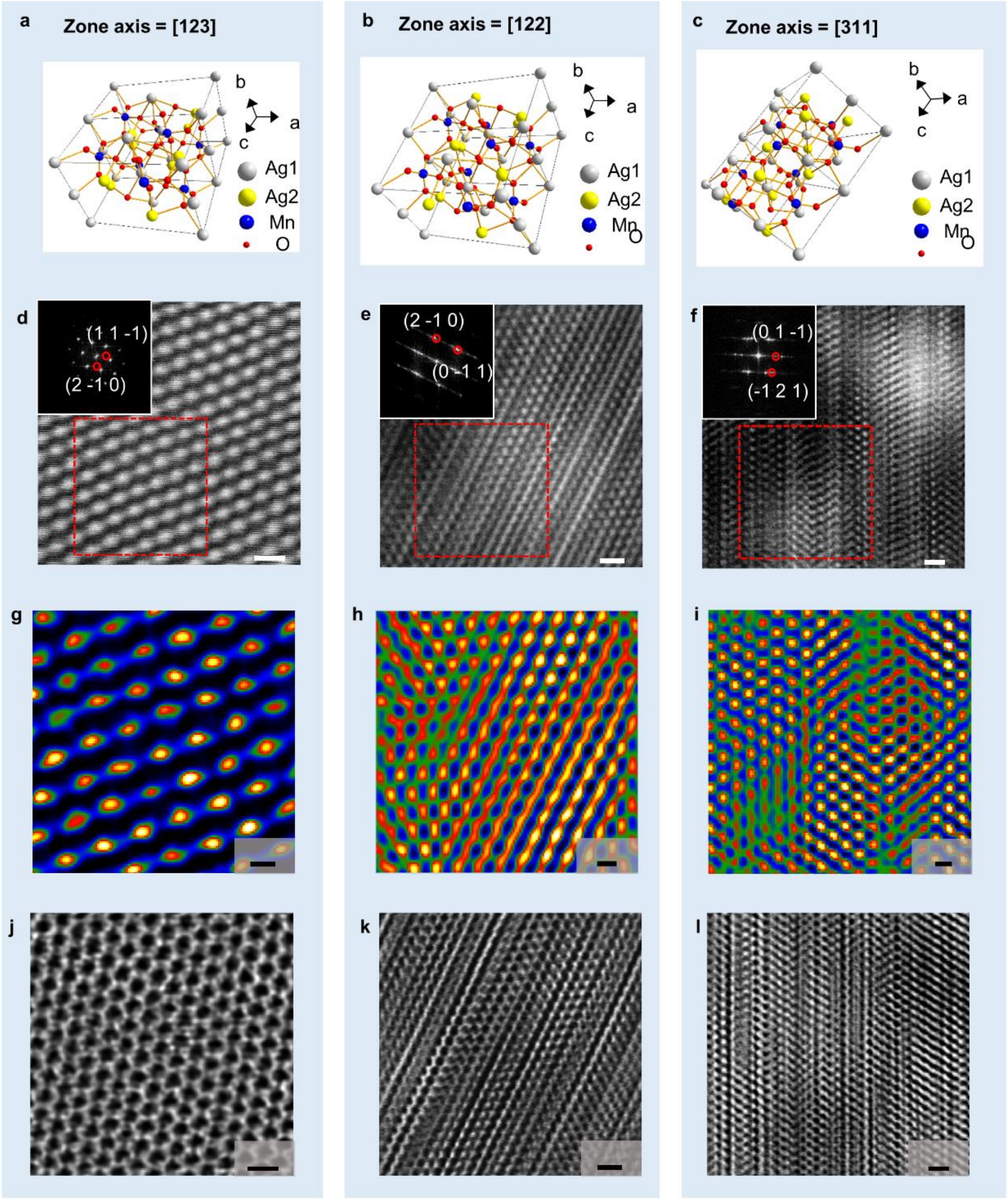

Supplementary Figure 12 | Crystallographic model and HAADF/BF-STEM microscopy. **a**–**l**, Illustration of Ag_2MnO_4 along [123] (**a**), [122] (**b**) and [311] (**c**) zone axis directions, showing complex orthrombic structure, and their filtered HAADF-STEM (**d–f**), simulated HAADF-STEM (**g–i**) and BF-STEM images (**j–l**). Scale bars, 0.5 nm (**d**–**f**), 0.2 nm (**g**–**i**), and 0.5 nm (**j**–**l**).

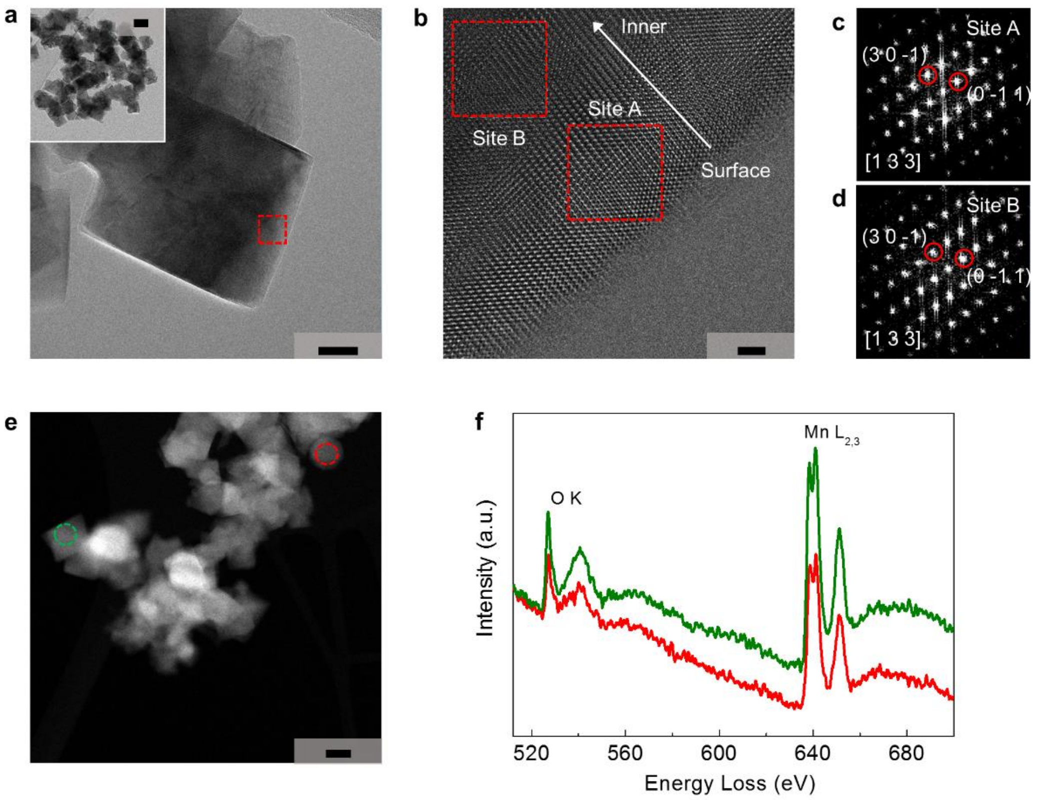

Supplementary Figure 13 | Electron microscopy and spectroscopy of the PM. a, HR-TEM image of the PM, showing nano-sized polyhedron structures. Inset image indicates low-magnified image (Scale bar, 100 nm). **b**–**d**, High-magnified image (**b**) of red box in **a**, and its fast Fourier transform patterns from surface (**c**) to inner (**d**) parts, showing Mn_3O_4 tetragonal crystal structure with no phase transition. **e**–**f**, HAADF-STEM image (**e**) and corresponding EELS spectra (**f**) at the marked red and green circles. Scale bars, 20 nm (**a**), 2 nm (**b**), and 10 nm (**e**).

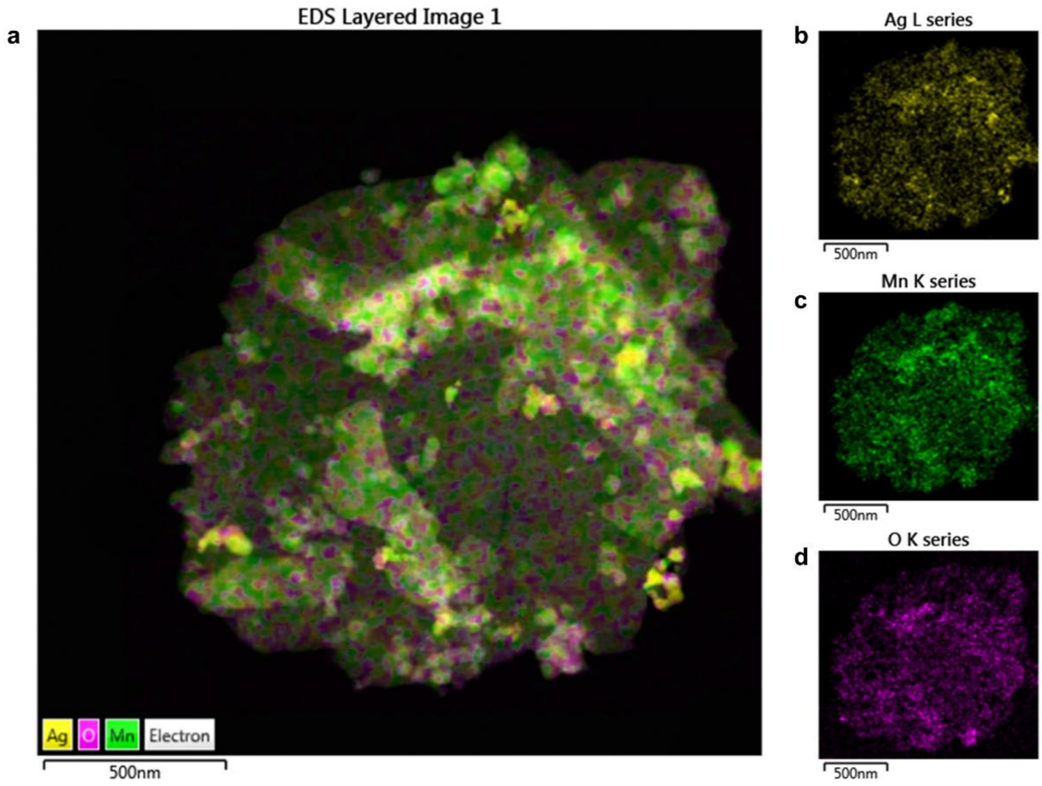

Supplementary Figure 14 | a–d, STEM-EDX mapping images of the SMNp (**a**), showing silver (**b**), manganese (**c**) and oxygen elements (**d**).

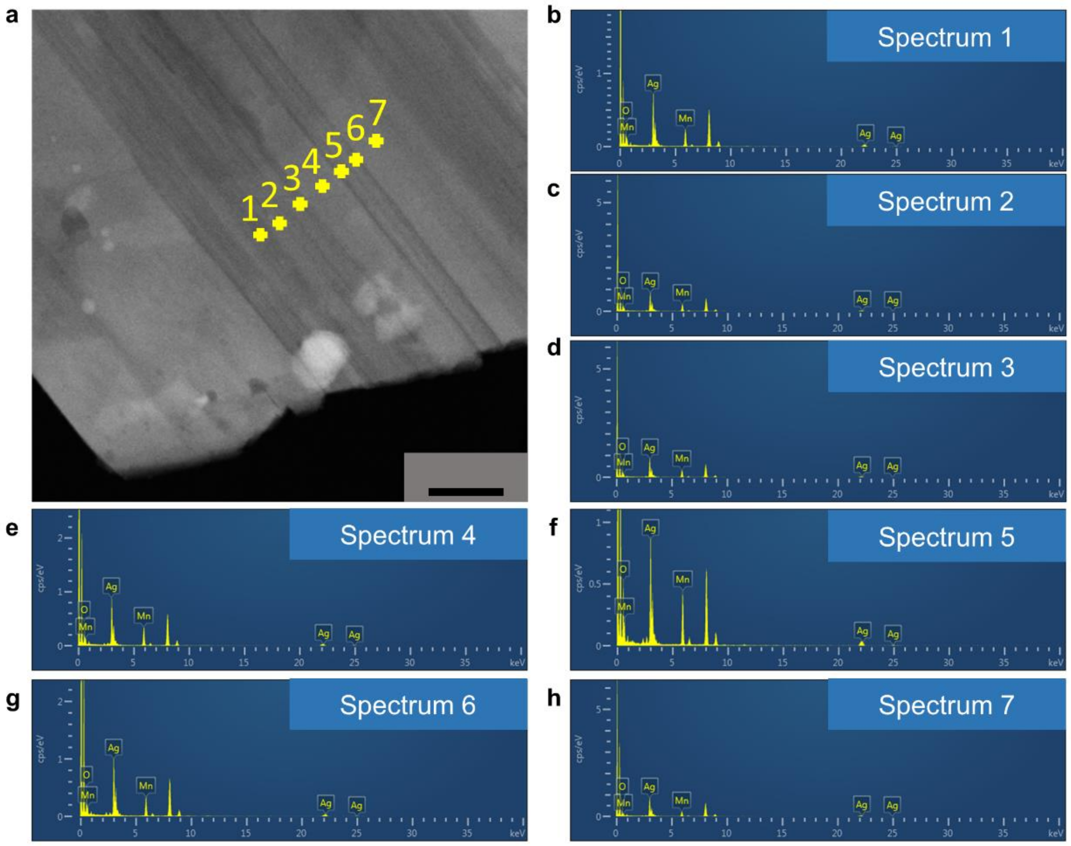

Supplementary Figure 15 | a–h, STEM image (**a**) and STEM-EDX spectra (**b−h**) of the SMNp at different points, showing silver, manganese and oxygen elements. Scale bar, 50 nm (**a**).

Supplementary Table 2 | Summary of point spectrum. Percent composition and atomic ratios of silver and manganese in the SMNp at different points in Supplementary Fig. 15, showing the molar ratio of ~2 (Ag/Mn).

Sites	Ag		Mn	
	**wt.%**	**at.%**	**wt.%**	**at.%**
1	72.25	39.55	16.04	17.24
2	68.10	34.74	18.23	18.26
3	70.79	35.46	14.24	14.00
4	67.67	34.42	18.63	18.61
5	64.89	32.87	21.80	21.68
6	68.63	34.97	17.55	17.56
7	70.17	34.41	14.10	13.58
Average at.%	35.20		17.28	
Molar ratio	2.04		1.00	

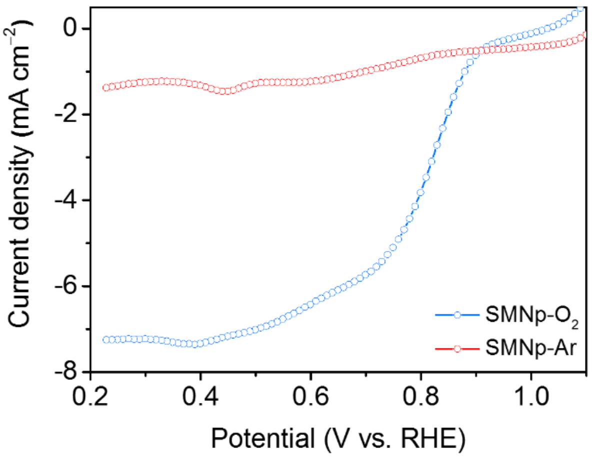

Supplementary Figure 16 | Capacitance correction for SMNp. Linear scan voltammorgram (LSV) curves for the SMNp in O_2 (blue) or Ar (red) saturated 0.1 M KOH solution with scan rate of 5 mV s^{-1}.

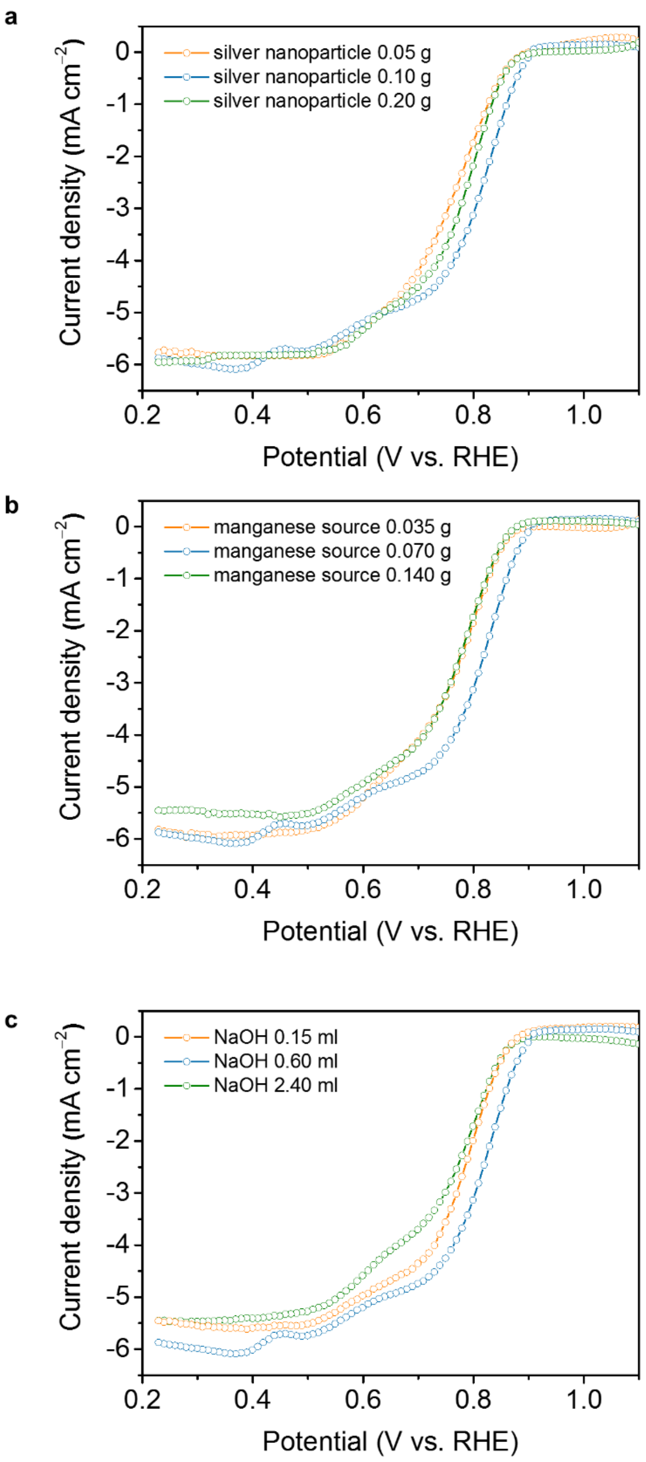

Supplementary Figure 17 | Oxygen reduction reaction activities of the control experiment. a–c, Linear scan voltammogram (LSV) curves for the control experiment with different amounts of Ag nanoparticles (**a**), manganese (**b**), and NaOH (**c**) at a RRDE (1,600

r.p.m) in O_2-saturated 0.1 M KOH solution with scan rate of 5 mV s^{-1}. All data have been calibrated by using Ar-saturated 0.1 M KOH solution.

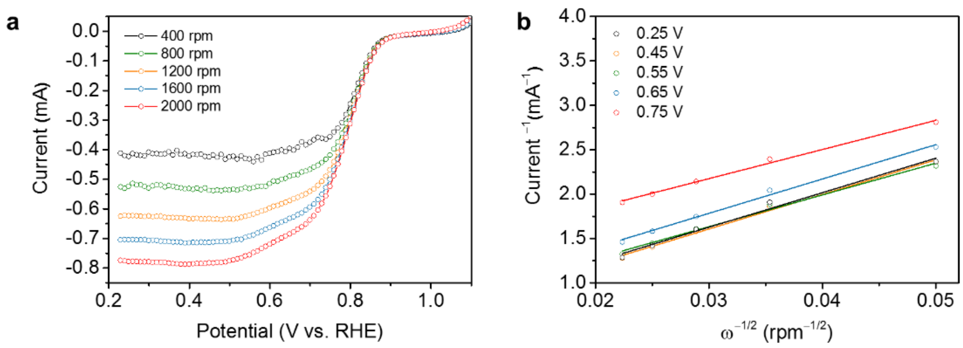

Supplementary Figure 18 | a,**b**, Linear scan voltammogram (LSV) curves (**a**) for the SMNp at the different rotation speed in O_2-saturated 0.1 M KOH solution with scan rate of 5 mV s^{-1} and corresponding Koutecky−Levich plots (**b**) at different potentials, indicating first order reaction with respect to the concentration of dissolved oxygen.

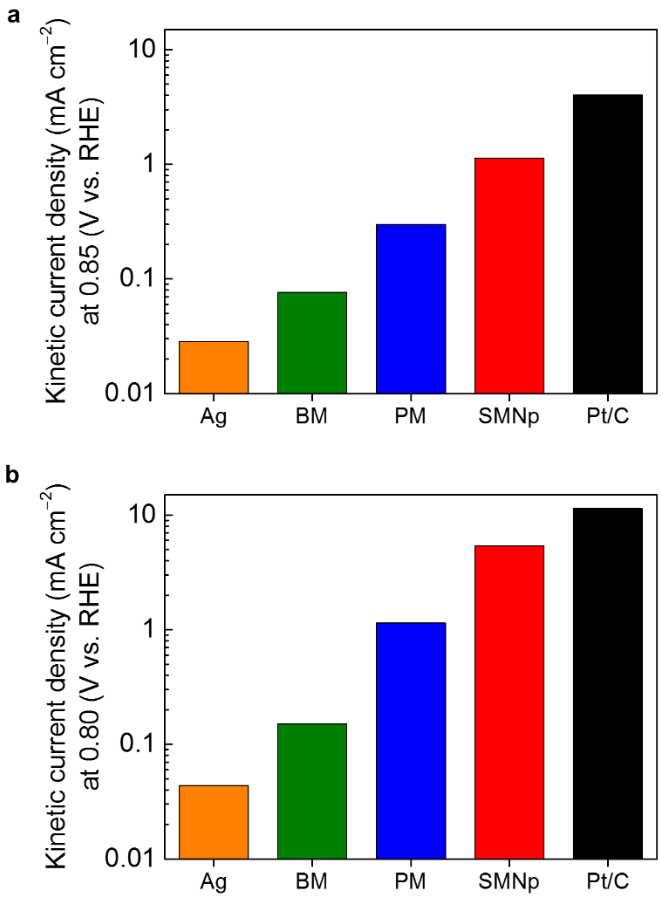

Supplementary Figure 19 | a,**b**, Kinetic current density of the Ag, BM, PM, SMNp and Pt/C at 0.85 V (**a**) and 0.80 V (**b**).

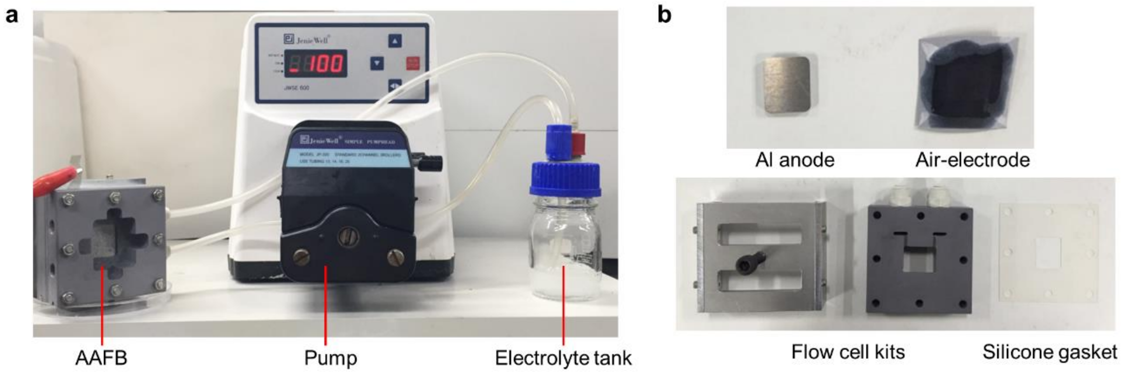

Supplementary Figure 20 | Components of the aluminum-air flow battery. a–b, Digital photograph of aluminum-air flow battery system (**a**) including a single stack cell, 6 M of KOH electrolytes and flow pump at 100 mL min^{-1}, and details of flow cell components (**b**).

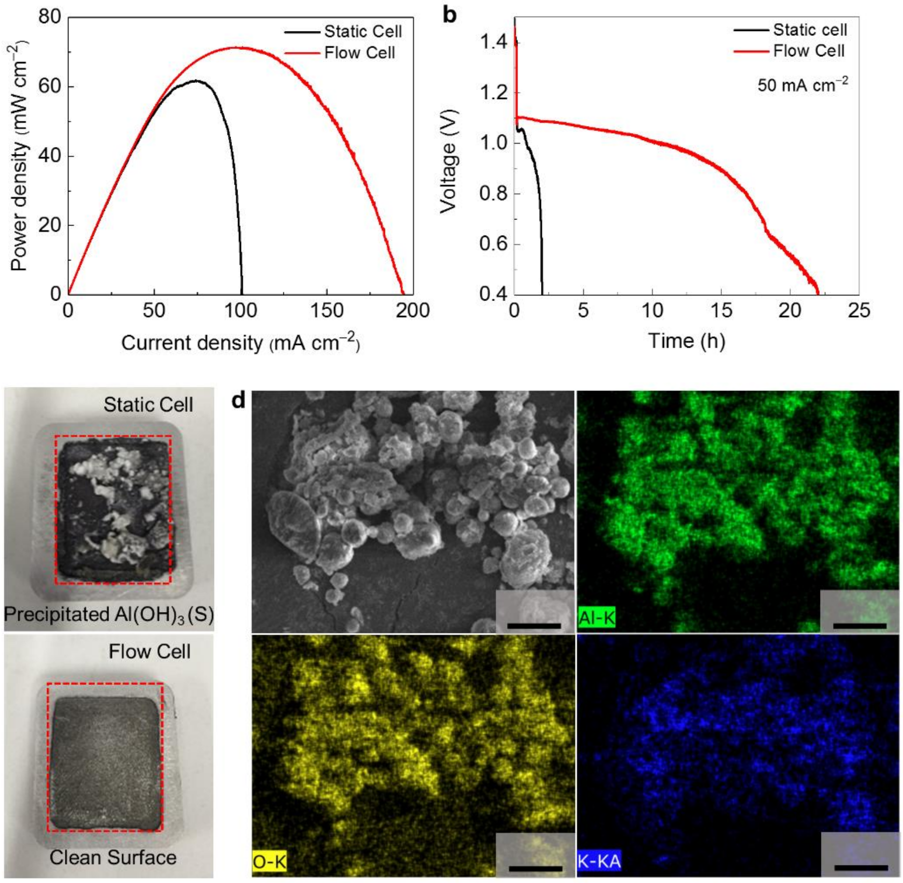

Supplementary Figure 21 | a,**b**, Power density curves (**a**) and discharge curves (**b**) of static and flow cell by using pristine air-electrode at 50 mA cm^{-2}. **c**, Photographs of aluminum anodes from discharged cells in **b**, showing the severe by-product formation on the surface of anode in static cell. **d**, SEM-EDX images of the byproducts on the anode of static cell after fully discharge process. Scale bars, 10 μm (**d**).

Supplementary Table 3 | Summary of power density for the primary aluminum-air flow batteries using all samples.

Sample	Air-electrode	Ag	BM	PM	SMNp	Pt/C
Peak power densities (mW cm^{-2})	71.3	82.1	85.1	88.2	105.2	104.0
Maximum current densities (mA cm^{-2})	195	210	205	220	237	226

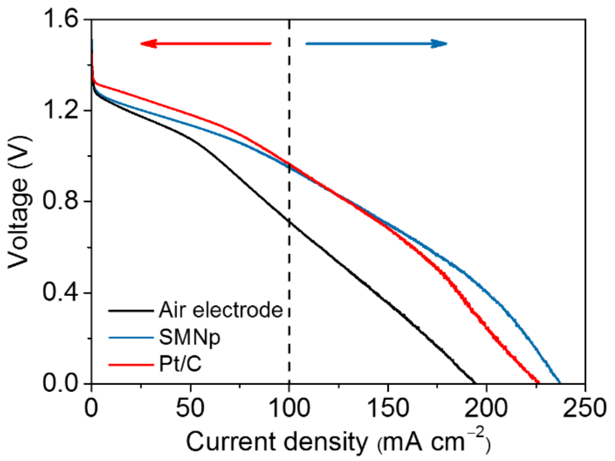

Supplementary Figure 22 | Polarization curves of aluminum-air flow batteries using the pristine air-electrode, SMNp and Pt/C with 6 M KOH electrolyte (scan rate of 0.1 mA s^{-1}) in Figure 4b.

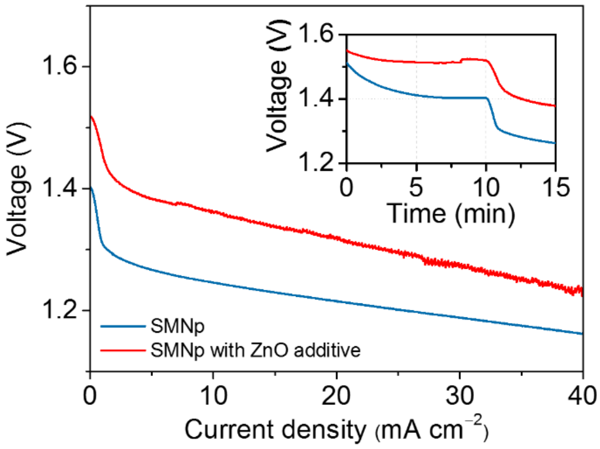

Supplementary Figure 23 | Polarization curves of aluminum-air flow batteries using the SMNp and SMNp with ZnO additive in 6M KOH electrolyte (scan rate of 0.1 mA s^{-1}). Inset image shows the OCP at rest condition.

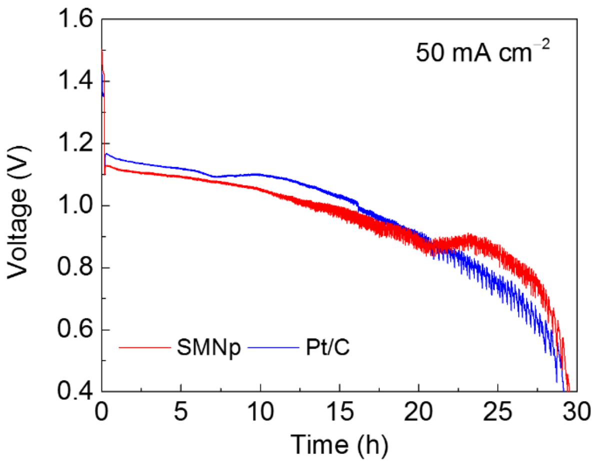

Supplementary Figure 24 | Discharge curves of the primary aluminum-air flow batteries using the SMNp and Pt/C at a current density of 50 mA cm^{-2}.

Supplementary Table 4 | Summary of electrochemical performance of the primary aluminum-air flow batteries using the pristine air-electrode, SMNp and Pt/C.

**Sample**	**Air-electrode**		**SMNp**		**Pt/C**	
**Current density ($mA\ cm^{-2}$)**	**50**	**100**	**50**	**100**	**50**	**100**
Discharge time (h)	22.0	5.8	29.5	7.3	29.1	7.5
Discharge capacity (mAh)	5,434	2,796	7,276	3,567	7,183	3,667
Specific capacity ($Ah\ kg^{-1}$)	2,107	2,702	2,642	2,843	2,538	2,859
Discharge energy (mWh)	4,965	2,140	6,998	3,202	6,965	3,215
Gravimetric energy density ($Wh\ kg^{-1}$)	1,925	2,068	2,541	2,552	2,461	2,507
Volumetric energy density ($Wh\ l^{-1}$)	5,198	5,584	6,861	6,890	6,645	6,769
Consumed aluminum (g)	2.5790	1.0348	2.7540	1.2548	2.8302	1.2826

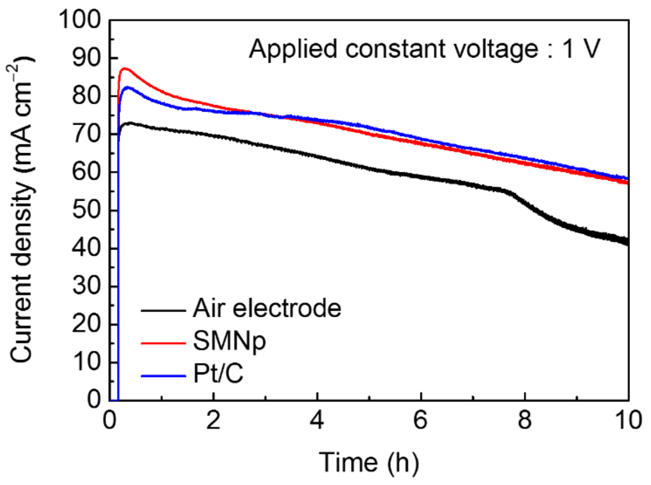

Supplementary Figure 25 | Current density behaviour of the Air electrode, SMNp and Pt/C at applied constant voltage of 1.0 V.

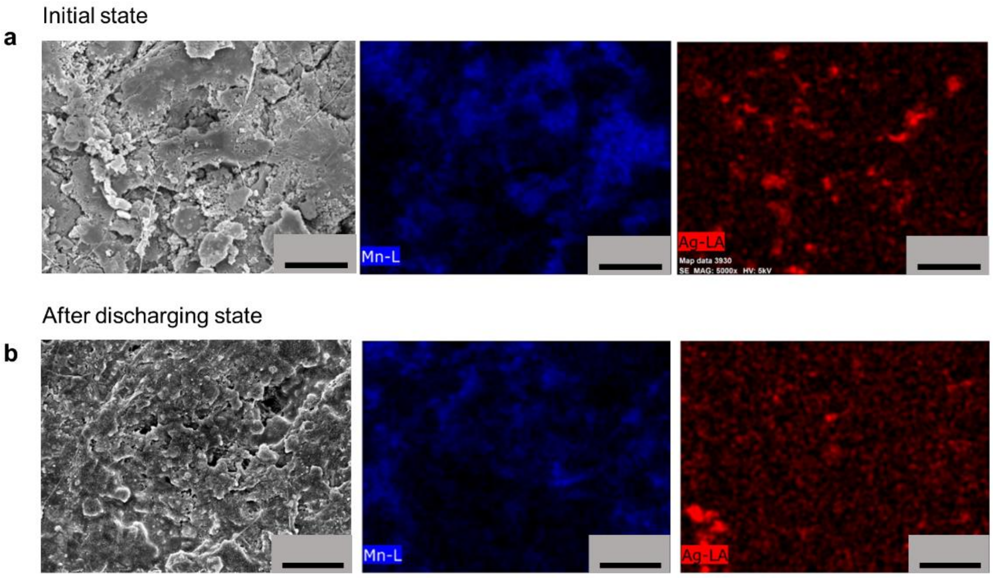

Supplementary Figure 26 | a,**b**, SEM-EDX images of the SMNp showing initial (**a**) and after discharging state (**b**). Scale bars, 6 μm (**a**,**b**).

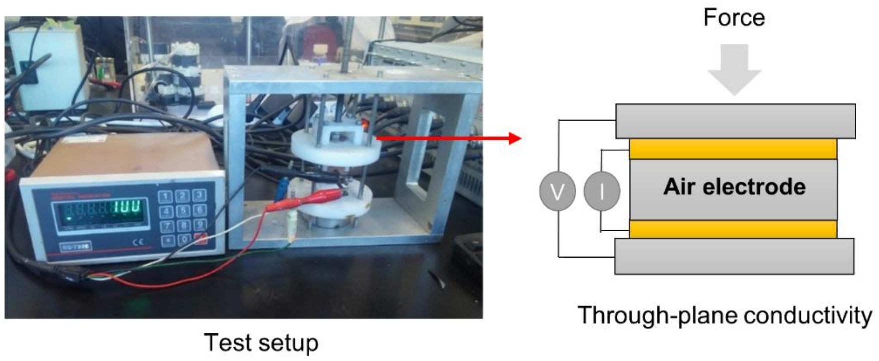

Supplementary Figure 27 | Electrical conductivity measurement. Digital photograph of electric device for checking resistivity of the air electrodes.

Supplementary Table 5 | Resistivty and conductivity of the electrocatalyst loaded air electrodes.

Sample	Air-electrode	Ag	BM	PM	SMNp	Pt/C
Resistivity (Ω cm)	30.2	19.8	27.6	23.7	19.3	22.2
Conductivity (S cm^{-1})	3.31×10^{-2}	5.05×10^{-2}	3.62×10^{-2}	4.23×10^{-2}	5.18×10^{-2}	4.5×10^{-2}

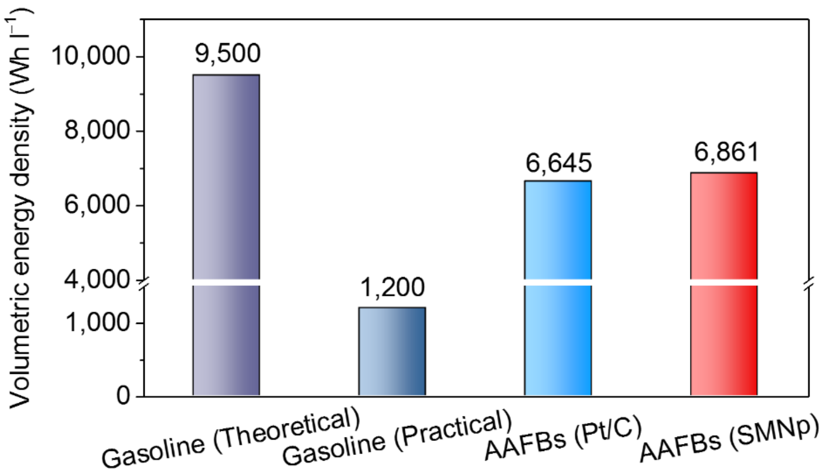

Supplementary Figure 28 | Comparison of the volumetric energy density among gasoline with theoretical and practical value, and AAFBs with Pt/C and SMNp (at 50 mA cm^{-2}).

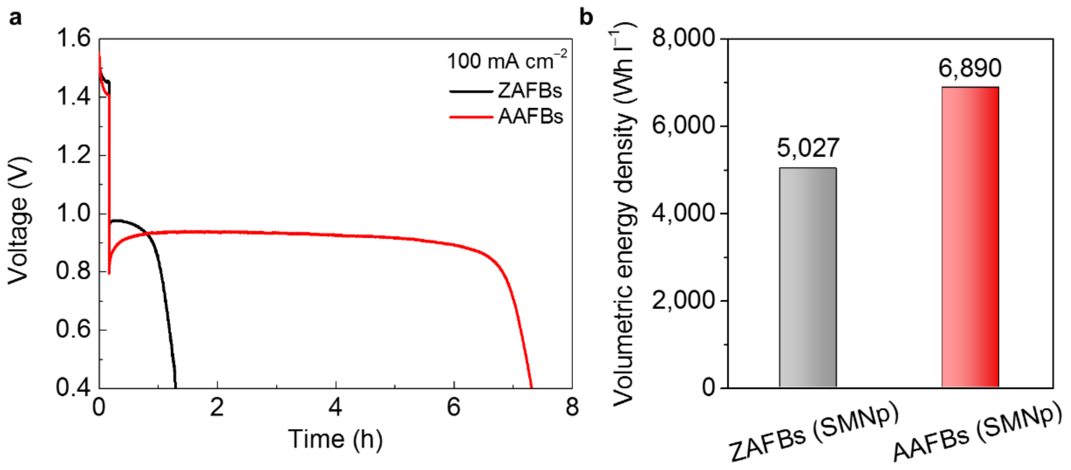

Supplementary Figure 29 | Comparision of Zn and Al-air flow batteries. a,**b**, Discharge curves of ZAFBs and AAFBs with the SMNp (**a**), and volumetric energy density (**b**) at a current density of 100 mA cm^{-2}.

Supplementary Table 6 | Summary of the electrochemical performance of the zinc and aluminum-air flow batteries using SMNp at current density of 100 mA cm^{-2}.

System	Zinc-air flow batteries	Aluminum-air flow batteries
Discharge time (h)	1.3	7.3
Discharge capacity (mAh)	558	3,567
Specific capacity (Ah kg^{-1})	804	2,843
Gravimetric energy density (Wh kg^{-1})	704	2,552
Volumetric energy density (Wh l^{-1})	5,027	6,890
Consumed metal (g)	0.6943	1.2548
Conversion efficiency (g Ah^{-1})	1.24	0.35

Supplementary Table 7 | Outline of reported electrocatalysts for the aluminum-air batteries with their electrochemical condition and performance.

Catalyst	Anode	Half-wave potential (V vs RHE)	Electrolyte		Average discharge voltage (V)	Current densities (mA cm^{-2})	Discharge time (h)	Peak power density (mW cm^{-2})	Specific capacity (Ah kg^{-1})	Gravimetric energy density (Wh kg^{-1})
			RDE	Cell						
**SMNp (This work)**	**Al alloy 6061 (97.93%)**	**0.80**	**0.1 M KOH**	**6 M KOH (Flowing)**	**1.10**	**50**	**29.5**	**105**	**2,642**	**2,541**
					**0.91**	**100**	**7.3**		**2,843**	**2,552**
CuNC/KB[1]	-	0.82	0.1 M KOH	6 M KOH + 0.01 M Na_2SnO_3 + 0.0005 M $In(OH)_3$ + 0.0075 M ZnO	1.53	40	20	-	-	-
Co_3O_4/N-KB[2]	-	0.80	0.1 M KOH	6 M KOH + 0.01 M Na_2SnO_3 + 0.0005 M $In(OH)_3$ + 0.0075 M ZnO	1.50	50	18	161	-	-
Ag/Fe_3O_4/N-KB[3]	-	0.82	0.1 M KOH	6 M KOH + 0.01 M Na_2SnO_3 + 0.0005 M $In(OH)_3$ + 0.0075 M ZnO	1.49	50	10	-	-	-
Co_3O_4-CeO_2/KB[4]		0.81	0.1 M KOH	6 M KOH + 0.01 M Na_2SnO_3 + 0.0005 M $In(OH)_3$ + 0.0075 M ZnO	1.27	50	10			
Co_3O_4/N-KB[5]	-	0.79	0.1 M KOH	6 M KOH + 0.01 M Na_2SnO_3 + 0.0005 M $In(OH)_3$ + 0.0075 M ZnO	1.52	20	15	-	-	-
MnO_2[6]	Al alloy (99.7%)	-	-	5 M KOH + Flax straw	1.0	25	6.5	-	-	-
MnO_2[7]	Al alloy 8011 (~98.32%)	-	-	4M KOH	1.30	10	3	13	-	816
					0.98	50	3	49	-	2,244
Ag/MnO_2[8]	Pure Al (99.99%)	0.68	0.1 M KOH	4 M KOH	1.34	100	24	204	-	-
Ag/CNT sheets[9]	Pure Al spring (99.999%)	-	-	PVA + PEO + KOH + ZnO + Na_2SnO_3 (hydrogel)	1.29	0.5	-	1.33	935	1,168
Co3O4/N-rGO/acetylene black[10]	10 h activation	0.83	0.1 M KOH	6 M KOH	1.19	300	40	357	-	-
Commercial procurement[11]	Grain size controlled pure Al	-	-	4 M NaOH	1.53	10	3	-	2,308	3,525

	(99.999%)									
60 wt% Pt/C[12]	Kitchen aluminum foil (97.60%)	-		3 M KOH-CH_3OH +3 M KOH-H_2O (dual electrolyte system)	1.15	20	-	28	1,810	2,081
$NiCo_2O_4$/CNTs[13]	-	-	0.1 M KOH	6 M KOH + 0.01 M Na_2SnO_3 + 0.0005 M $In(OH)_3$ + 0.0075 M ZnO	1.20	200	20	-	-	-

Supplementary references

1 Li, J. *et al.* Cu-MOF-derived Cu/Cu_2O nanoparticles and cunxcy species to boost oxygen reduction activity of ketjenblack carbon in al-air battery. *ACS Sustain. Chem. Eng.* **6**, 413-421, (2018).

2 Li, J. *et al.* Co_3O_4/Co-N-C modified ketjenblack carbon as an advanced electrocatalyst for al-air batteries. *J. Power Sources* **343**, 30-38, (2017).

3 Li, F. *et al.* Ag/Fe_3O_4-N-doped ketjenblack carbon composite as highly efficient oxygen reduction catalyst in al-air batteries. *J. Electrochem. Soc.* **164**, A3595-A3601, (2017).

4 Liu, K. *et al.* Co_3O_4–CeO_2/C as a highly active electrocatalyst for oxygen reduction reaction in al–air batteries. *ACS Appl. Mater. Interfaces* **8**, 34422-34430, (2016).

5 Liu, K. *et al.* N-doped carbon supported Co_3O_4 nanoparticles as an advanced electrocatalyst for the oxygen reduction reaction in al-air batteries. *RSC Adv.* **6**, 55552-55559, (2016).

6 Grishina, E. *et al.* Improvement of aluminum–air battery performances by the application of flax straw extract. *ChemSusChem* **9**, 2103-2111, (2016).

7 Fan, L., Lu, H., Leng, J., Sun, Z. & Chen, C. The study of industrial aluminum alloy as anodes for aluminum-air batteries in alkaline electrolytes. *J. Electrochem. Soc.* **163**, A8-A12, (2016).

8 Sun, S. *et al.* Oxygen reduction reaction catalysts of manganese oxide decorated by silver nanoparticles for aluminum-air batteries. *Electrochim. Acta* **214**, 49-55, (2016).

9 Xu, Y., Zhao, Y., Ren, J., Zhang, Y. & Peng, H. An all-solid-state fiber-shaped aluminum-air battery with flexibility, stretchability, and high electrochemical performance. *Angew. Chem. Int. Ed.* **55**, 7979-7982, (2016).

10 Zhang, Y. *et al.* Facile fabrication of sandwich-structured Co_3O_4/N-rGO/AB hybrid with enhanced orr electrocatalytic performances for metal-air batteries. *RSC Adv.* **5**, 9057-9063, (2015).

11 Fan, L. & Lu, H. The effect of grain size on aluminum anodes for al–air batteries in alkaline electrolytes. *J. Power Sources* **284**, 409-415, (2015).

12 Wang, L. *et al.* A high-capacity dual-electrolyte aluminum/air electrochemical cell. *RSC Adv.* **4**, 30857-30863, (2014).

13 Zhang, H. *et al.* Nickel cobalt oxide/carbon nanotubes hybrid as a high-performance electrocatalyst for metal/air battery. *Nanoscale* **6**, 10235-10242, (2014).
