# Supplementary material for: Seed-mediated atomic-scale reconstruction of silver manganate nanoplates for oxygen reduction towards high-energy aluminum-air flow batteries
Source: Nat Commun. 2018 Sep 13;9:3715. doi: 10.1038/s41467-018-06211-3 (PMC6137061; doi:10.1038/s41467-018-06211-3)
Supplement: Supplementary file 2 — Peer Review File [file 41467_2018_6211_MOESM2_ESM.pdf]

Editorial Note: This manuscript has been previously reviewed at another journal that is not operating a transparent peer review scheme. The manuscript was considered suitable for publication without further review at *Nature Communications.*
